# ACAT1‐Mediated SP1‐K694 Lactylation Promotes M1 Macrophage Polarization via TREM1 Transcription in COPD

**DOI:** 10.1002/advs.78021

**Published:** 2026-09-27

**Authors:** Ling Lin, Ping Zhang, Qing Song, Tao Li, Yuqin Zeng, Ping Chen

**Affiliations:** ^1^ Department of Respiratory and Critical Care Medicine The Second Xiangya Hospital Central South University Changsha Hunan China; ^2^ Clinical Medical Research Center for Pulmonary and Critical Care Medicine in Hunan Province Changsha Hunan China; ^3^ Diagnosis and Treatment Center of Respiratory Disease Central South University Changsha Hunan China; ^4^ Research Unit of Respiratory Disease Central South University Changsha Hunan China

**Keywords:** ACAT1, airway inflammation, chronic obstructive pulmonary disease, macrophage polarization, SP1‐K694la

## Abstract

Aberrant macrophage polarization is one of the key mechanisms underlying chronic obstructive pulmonary disease (COPD), but the underlying processes have not yet been fully elucidated. Lactylation is a novel lactate‐derived post‐translational modification and is relatively active in M1 macrophages. This study identified SP1 lactylation at lysine 694 (SP1‐K694la), which was elevated in Cigarette Smoke Extract(CSE)‐induced macrophages and COPD mice and enhanced SP1 enrichment at the TREM1 promoter. TREM1 knockdown reduced M1 macrophages. The SP1‐K694R mutation (mimicking delactylation) decreased SP1‐K694la, reduced SP1 enrichment at the TREM1 promoter, and downregulated M1 macrophages under CSE treatment. In contrast, the SP1‐K694Q mutation (mimicking constitutive lactylation) increased SP1‐K694la, enhanced SP1 enrichment at the TREM1 promoter, and promoted M1 macrophage polarization. Furthermore, TREM1 knockdown alleviated SP1‐K694la‐mediated increase in M1 macrophages and reduced secretion of IL‐1β, IL‐6, and TNF‐α. In addition, ACAT1 was identified as a lactyltransferase for SP1‐K694la. In COPD patients, lactate levels, Pan‐Kla, LDHA, SP1‐K694la, and TREM1 were elevated. Lactate levels correlated positively with smoking index and were higher in GOLD 3–4 than in GOLD 1 patients, while TREM1 mRNA correlated negatively with FEV1, FEV1%pred, and FEV1/FVC.ACAT1 acts as a lactyltransferase, increasing SP1‐K694la to enhance TREM1 transcription, which promotes M1 polarization and airway inflammation in COPD.

## Graphical abstract

Upon cigarette smoke exposure(CSE), LDHA expression is increased in lung macrophages, promoting the conversion of pyruvate to lactate and elevating the lactylation level of SP1 at the K694 site. This, in turn, enhances SP1 enrichment at the TREM1 promoter, increases TREM1 expression, promoted M1 macrophage polarization, and triggers the secretion of pro‐inflammatory cytokines IL‐1β, IL‐6, and TNF‐α, leading to airway inflammation and ultimately contributing to the development of COPD. Besides, intervention with an LDHA inhibitor, Oxamate, reduces lactate levels, thereby attenuating SP1‐K694 lactylation and M1 macrophage polarization. Furthermore, CSE also increased ACAT1 expression in macrophages, and ACAT1 functions as a lactyltransferase that increases SP1‐K694 lactylation.

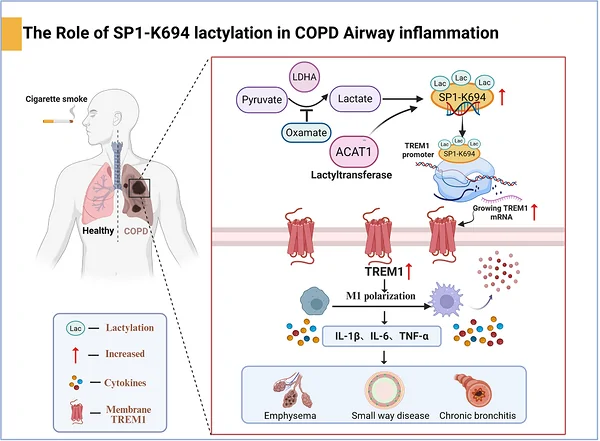

**Abbreviations**
ACAT1: acetyl‐CoA acetyltransferase 1
BMDM: Bone marrow‐derived macrophages
COPD: Chronic obstructive pulmonary disease
CS: Cigarette smoke
CSE: Cigarette smoke extract
DAPI: 4’,6‐diamidino‐2’‐phenylindole
DI: Destructive index
DMEM: Dulbecco's Modified Eagle Medium
ELISA: Enzyme linked immunosorbent assay
FEV1: Forced expiratory volume in 1 s
FEV100: Forced expiratory volume in 100 s
FVC: Forced vital capacity
MLI: Mean linear intercept
PEF: Peak expiratory flow
RT‐qPCR: Reverse transcription quantitative polymerase chain reaction
SP1: Specificity Protein 1
TI/TE: Inspiratory time/expiratory time
TREM1: Triggering receptor expressed on myeloid cells 1

## 1 Introduction

Chronic obstructive pulmonary disease (COPD) is characterized by persistent dyspnoea and chronic airway inflammation, which progressively destroys the lung parenchyma and airway structure, resulting in markedly irreversible and progressive airflow limitation [1, 2]. Globally, COPD affects more than 400 million individuals and ranks as the third leading cause of death worldwide, with approximately 16.9 million new cases and 3.3 million deaths reported annually [3]. In recent decades, COPD has emerged as a major public health concern worldwide [4]. Therefore, there is an urgent need to investigate the underlying pathogenesis and identify critical targets for intervention.

Cigarette smoke, the main risk factor for COPD, triggers a cascade of cellular events, including inflammation, oxidative stress, and apoptosis [5, 6]. These processes result in cell injury and death, structural destruction of lung tissue, loss of pulmonary function, and further acceleration of disease progression [7]. Pulmonary macrophages serve as the first line of defence against inhaled particulates and pathogens in the lung, while also producing and releasing a variety of proinflammatory mediators that trigger pulmonary inflammatory responses [8]. Under cigarette smoke exposure, macrophages secrete a range of cytokines, chemokines, and growth factors (interleukin [IL]‐1β, IL‐6, tumor necrosis factor‐α [TNF‐α], CXCL2, matrix metalloproteinase‐9, etc.), which compromise the pulmonary epithelial and endothelial barrier [9]. Macrophages exist in two main phenotypes, M1 and M2, and aberrant macrophage polarization is a key mechanism in COPD progression. M1 macrophages are primarily involved in airway inflammation and tissue destruction, whereas M2 macrophages predominantly appear in the later stages of the disease and participate in lung structural remodeling [10]. Studies have shown that increased M1 macrophages contribute to heightened airway inflammation in COPD [11].

Lactate is a key carbon‐containing metabolite derived from glycolysis and acts as a substrate mediating lactylation at specific lysine residues of proteins [12]. Lactate dehydrogenase A (LDHA) is a key enzyme that converts pyruvate to lactate during glycolysis [13]. Early studies on lactylation identified this modification during M1 macrophage polarization, in which increased lactylation was observed [12]. Due to enhanced glycolysis and increased lactate levels in M1 macrophages, lactylation is relatively active [14]. Accumulating evidence indicates that lactylation not only regulates M2 macrophages to facilitate tumor immune evasion [12, 15], but also participates in M1 macrophage polarization [16]. Furthermore, a study by Chen et al. [17]. revealed that lactate levels, as well as the protein expression of LDHA and pan‐lysine lactylation (Pan‐Kla), are elevated in patients with COPD. Histone lactylation (e.g., H3K14la and H4K12la) has been studied in COPD models induced by polycyclic aromatic hydrocarbons [17], such as benzo[a]pyrene and 1‐nitropyrene [18], whereas protein lactylation in COPD models directly induced by cigarette smoke remains to be elucidated. Besides, the functional significance of non‐histone protein lactylation in COPD remains entirely unexplored. Unlike histone lactylation, which modulates gene transcription indirectly through chromatin remodelling [12], non‐histone lactylation modifies the functional properties of target proteins, including their stability, enzymatic activity, and protein‐protein interactions, thereby enabling rapid and direct regulation of cellular signalling [19, 20]. Emerging evidence has demonstrated that non‐histone lactylation actively participates in the regulation of macrophage polarization and regulatory Treg cell function [16, 21].

In the preliminary phase of this study, integrated proteomic and lactylation proteomic sequencing was performed to identify lactylation modifications in cigarette smoke extract (CSE)‐treated macrophages. The results revealed an elevated lactylation level at the K694 residue of the transcription factor specificity protein 1 (SP1), a non‐histone protein [22]. Recent studies have established SP1 as a critical regulator of macrophage functions and lung inflammation [23]. Specifically, SP1 transcriptionally regulates Prohibitin 1 (PHB1), driving M1 macrophage polarization while impairing phagocytic function, thereby exacerbating acute lung injury [23]. Given that lactylation of transcription factors has been shown to regulate target gene transcription [24], whether SP1 lactylation influences macrophage polarization through modulation of its downstream targets in COPD remains unclear.

The objectives of this study were to elucidate the role and underlying mechanism of SP1 lactylation in pulmonary macrophage polarization in COPD and to further explore the lactyltransferase that regulates SP1 lactylation levels.

Herein, we confirmed that lactate levels and SP1 lactylation at lysine K694 (SP1‐K694la) are increased in cigarette smoke‐induced COPD. Mechanistically, SP1‐K694la mediates transcriptional activation of triggering receptor expressed on myeloid cells 1 (TREM1), thereby driving macrophage M1 polarization and subsequent IL‐1β, IL‐6, and TNF‐α secretion, which activates airway inflammation in COPD. Furthermore, we identified acetyl‐CoA acetyltransferase 1 (ACAT1) as a lactyltransferase involved in SP1‐K694 lactylation in CSE‐induced macrophages. These findings uncover a novel mechanism involving the ACAT1/SP1‐K694/TREM1 axis in COPD, offering an actionable target for therapeutic intervention.

## 2 Results

### 2.1 Role of Lactylation in Cigarette Smoke–Induced COPD

**FIGURE 1:**
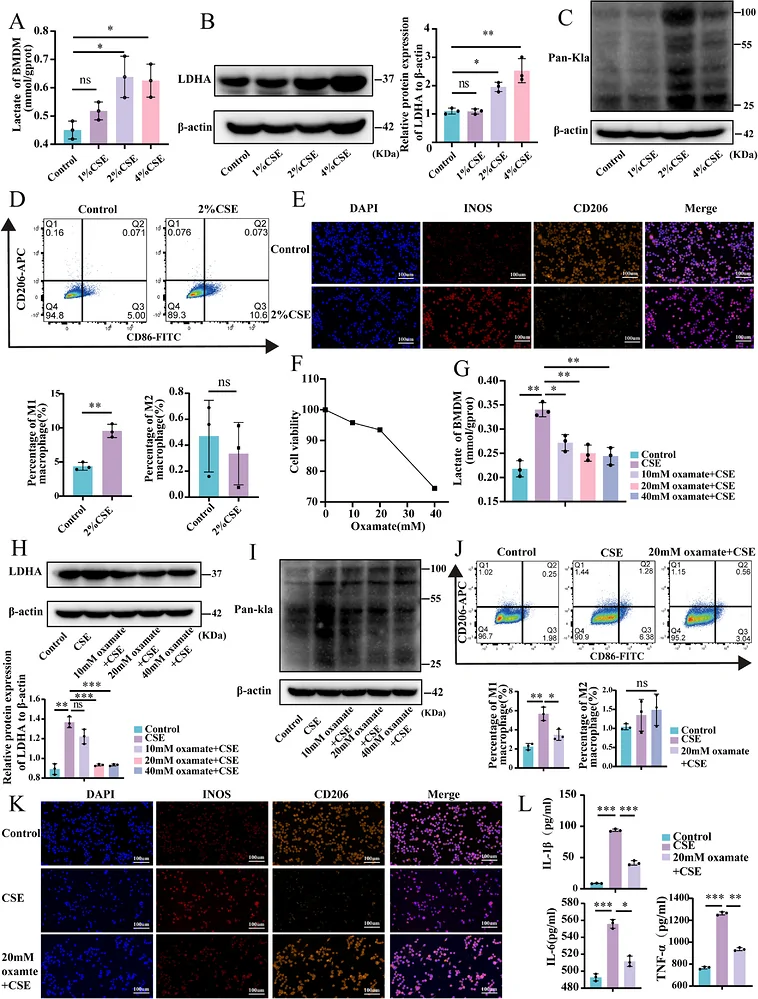
Lactylation levels are associated with CSE‐induced macrophage polarization. (A) Lactate production in BMDMs treated with 0%, 1%, 2%, and 4% CSE (*n* = 3 independent experiments). (B) LDHA expression in BMDMs treated with different CSE concentrations (0%, 1%, 2%, and 4%), analyzed by western blotting (*n* = 3 per group). (C) Pan‐Kla expression in BMDMs treated with different CSE concentrations (0%, 1%, 2%, and 4%), analyzed by western blotting (*n* = 3 per group). (D) Expression of the M1 macrophage marker CD86 and the M2 macrophage marker CD206 in CSE‐induced BMDMs, detected by flow cytometry. (E) IF detection of the M1 macrophage marker iNOS (red) and the M2 macrophage marker CD206 (orange) in CSE‐induced BMDMs (scale bar = 100 µm). (F) Cell viability of BMDMs treated with different concentrations of oxamate (0, 10, 20, and 40 mm) assessed using the Cell Counting Kit‐8. (G) Lactate production (H), LDHA expression, and (I) Pan‐Kla expression in CSE‐induced BMDMs treated with different concentrations of oxamate (0, 10, 20, and 40 mm). (J) CD86 and CD206 expression in CSE‐induced BMDMs treated with 20 mm oxamate, detected by flow cytometry. (K) iNOS (red) and CD206 (orange) expression in CSE‐induced BMDMs treated with 20 mm oxamate, detected by IF (scale bar = 100 µm). (L) Concentrations of IL‐1β, IL‐6, and TNF‐α in the supernatant of CSE‐induced BMDMs treated with 20 mm oxamate, measured by enzyme‐linked immunosorbent assay. CSE, 2.5% CSE. *t*‐test (2 groups) or ANOVA+Tukey (≥3 groups) were used.^*^ *p* < 0.05, ^**^ *p* < 0.01, ^***^ *p* < 0.001.

Bone barrow‐derived macrophages (BMDMs)Deoxyribonucleic Acid were isolated from normal mice and cultured. Flow cytometric analysis revealed that 93.66% of the cells were F4/80^+^ and CD11b^+^ double‐positive macrophages (Figure S1A). After the BMDMs were treated with different concentrations of CSE (0%, 1%, 2%and 4%), lactate production and LDHA protein expression were elevated in CSE‐treated BMDMs (Figure 1A,B). In addition, the level of Pan‐Kla was increased in BMDMs treated with 2% and 4% CSE (Figure 1C). Considering that Pan‐Kla levels were higher in BMDMs treated with 2% CSE than with 4% CSE, 2% CSE was selected for subsequent cell experiments. Both flow cytometric analysis and immunofluorescence (IF) staining showed that the proportion of M1 macrophages (CD86^+^) was increased in BMDMs exposed to CSE (Figure 1D,E).

We treated the BMDMs with different concentrations of oxamate (10, 20, and 40 mm) to modulate lactate levels. The lactate concentration and the protein expression of LDHA and Pan‐Kla were reduced after oxamate treatment (Figure 1G–I). Because cell viability was significantly impaired at 40 mm oxamate (Figure 1F), 20 mM was selected for subsequent experiments. In addition, the proportion of M1 macrophages was lower in the oxamate‐treated group than in the CSE group (Figure 1J,K). Enzyme‐linked immunosorbent assay(ELISA) demonstrated that the levels of IL‐1β, IL‐6, and TNF‐α in the cell supernatant were significantly reduced following oxamate treatment (Figure 1L).

**FIGURE 2:**
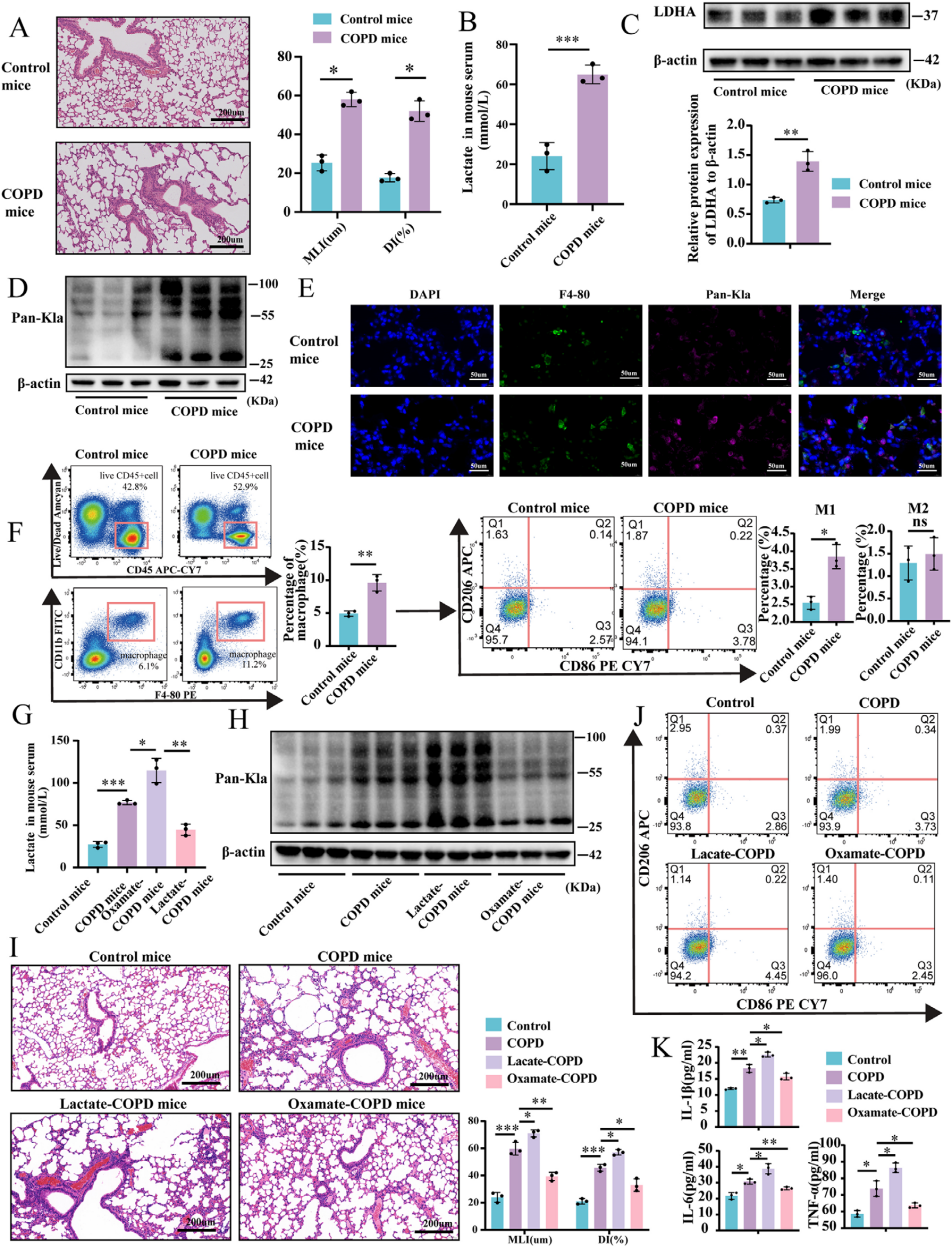
Lactylation levels are associated with macrophage polarization in COPD model mice. (A) H and E staining of mouse lung tissue (scale bar = 200 µm). (B) Lactate production, (C) LDHA expression, and (D) Pan‐Kla expression in the lungs of COPD model mice. (E) Colocalization of F4/80 and Pan‐Kla in the lungs of COPD mice. Tissue sections were co‐stained with anti‐F4/80 (green, macrophage marker) and anti‐Pan‐Kla (red). Nuclei were counterstained with DAPI (blue) (scale bar = 50 µm). (F) Flow cytometric analysis of lung macrophages (CD45^+^CD11b^+^F4/80^+^) and CD86/CD206 expression in COPD mice. Gating: viable cells → CD45^+^ → CD11b^+^F4/80^+^. Percentages of macrophages and CD86/CD206 levels (% or MFI) were compared. (G–I) Lactate concentrations in mouse serum, Pan‐Kla expression, and H&E staining of lung tissue in COPD model mice treated with L‐lactate (500 mg/kg) and sodium oxamate (500 mg/kg). (J) CD86 and CD206 expression in macrophages from digested lung tissues of COPD model mice treated with L‐lactate and sodium oxamate. (K) Concentrations of IL‐1β, IL‐6, and TNF‐α in BALF from COPD model mice treated with L‐lactate and sodium oxamate. *n* = 8 mice per group; COPD, chronic obstructive pulmonary disease; BALF, bronchoalveolar lavage fluid; DAPI, 4′,6‐diamidino‐2′‐phenylindole; DI, destructive index; MLI, mean linear intercept. ANOVA+Tukey were used,^*^ *p* < 0.05, ^**^ *p* < 0.01, ^***^ *p* < 0.001.

To investigate whether lactylation levels change in mice with COPD, we established a COPD mouse model. Compared with control mice, those in the COPD group showed enlarged alveolar spaces, destruction of alveolar walls, and severe airway inflammation cell infiltration(Figure 2A). Lactate levels in mouse serum, LDHA in lung tissue, as well as Pan‐Kla levels of lung macrophages, were increased in mice with COPD (Figure 2B–E). COPD model mice also exhibited an increased proportion of macrophages, along with a higher percentage of M1 macrophages (Figure 2F).

Furthermore, following L‐lactate administration, lactate levels in mouse serum and Pan‐Kla levels in lung tissue were upregulated. This was accompanied by worsened pulmonary function, aggravated emphysema and airway inflammation, an increased proportion of M1 macrophages, and elevated levels of IL‐1β, IL‐6, and TNF‐α in bronchoalveolar lavage fluid (BALF). By contrast, in the Oxamate‐COPD group, Oxamate administration decreased lactate levels in mouse serum and reduced Pan‐Kla levels in lung tissue. These changes were associated with improvements in pulmonary function and body weight, reductions in emphysema and airway inflammation, a lower proportion of M1 macrophages, and decreased levels of IL‐1β, IL‐6, and TNF‐α in BALF compared with the COPD group (Figure 2G–K and Figure S1B–E).

### 2.2 Lactylome Analysis in CSE‐Induced Macrophages

**FIGURE 3:**
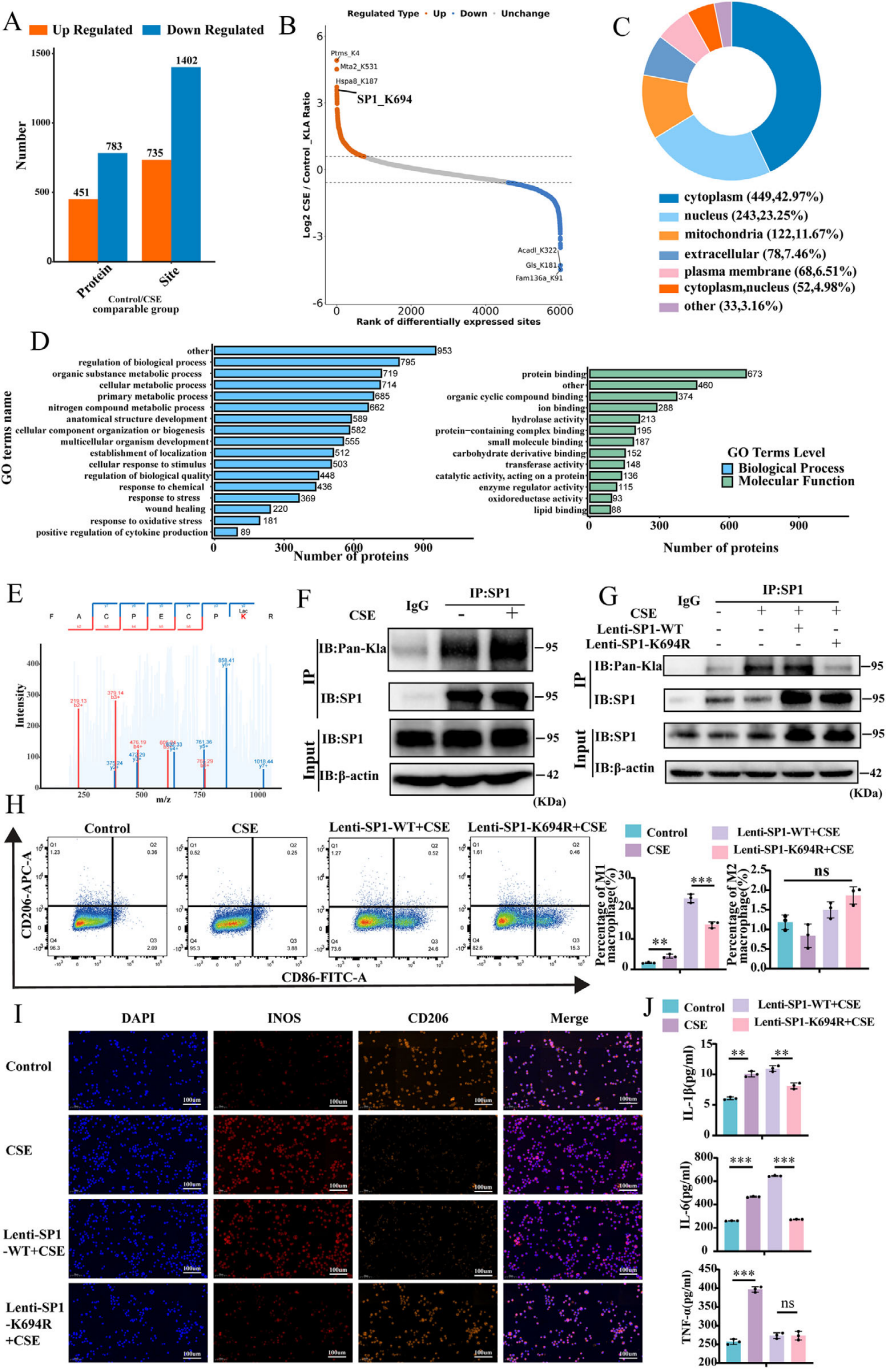
Lactylation of SP1 plays an important role in CSE‐induced M1 macrophage polarization. (A) Bar graph of omics data showing the number of differentially expressed lactylation‐modified proteins and lactylation sites identified by MS/MS. (B) Scatter plot of differentially modified lactylation sites identified by MS/MS. (C) Subcellular localization of DELPs shown as pie charts. (D) Enrichment analysis of DELPs based on the Gene Ontology database. (E) Collision‐induced dissociation (CID) analysis used to identify the modification site of SP1‐K694 by MS/MS spectrum. (F) SP1 lactylation levels in CSE‐induced BMDMs, detected by IP using an anti‐SP1 antibody or control IgG. (G) SP1 lactylation levels detected by IP in CSE‐induced MH‐S cells expressing the SP1‐K694R mutant. (H) CD86 and CD206 expression in CSE‐induced MH‐S cells expressing the SP1‐K694R mutant, detected by flow cytometry. (I) iNOS (red) and CD206 (orange) expression in CSE‐induced MH‐S cells expressing the SP1‐K694R mutant, detected by IF (scale bar = 100 µm). (J) Concentrations of IL‐1β, IL‐6, and TNF‐α in the supernatant of CSE‐induced MH‐S cells after transduction with the SP1‐K694R mutant. ANOVA+Tukey were used,^**^ *p* < 0.01, ^***^ *p* < 0.001.

To characterize the landscape of lactylation in CSE‐induced BMDMs, we performed lactylome analysis using a 4D label‐free platform to identify differentially lactylated proteins. In total, 6012 lactylated sites across 1777 proteins were identified in CSE‐induced macrophages (Figure S2A). Quality control indicated that the peptide distribution was within a reasonable range (Figure S2B). We identified 735 sites in 451 proteins with increased lactylation and 1402 sites in 735 proteins with decreased lactylation under CSE conditions, which were defined as differentially expressed lactylated proteins (DELPs) (Figure 3A,B). Among the identified proteins and sites, 360 sites in 111 proteins were classified as transcription factors (Figure S2C). Notably, Kla‐modified proteins were distributed not only in the cytoplasm but also in the nucleus, mitochondria, and plasma membrane (Figure 3C). Gene Ontology cluster analysis indicated that DELPs may play roles in macrophage polarization (including response to oxidative stress, wound healing, and positive regulation of cytokine production), as well as in Deoxyribonucleic Acid (DNA) and protein binding (Figure 3D).

### 2.3 Lactylation of SP1 Plays an Important Role in CSE‐Induced M1 Macrophage Polarization

Among these DELPs, SP1‐K694la was upregulated in CSE‐induced macrophages (Figure 3B), and SP1 is a multifunctional transcription factor. IF staining showed colocalization of the Kla modification and SP1 in macrophages (Figure S2E). Immunoprecipitation (IP) analysis confirmed that SP1 lactylation was upregulated in CSE‐treated BMDMs (Figure 3F). Moreover, SP1 protein expression remained unchanged following CSE treatment (Figure S2F). Proteomic analysis identified only one lactylated lysine residue in SP1 (K694) (Figure 3E).

Given the technical challenges and low transfection efficiency of both plasmid and viral transduction in BMDMs, Murine Alveolar Macrophage Cell Line (MH‐S) cells were used for subsequent experiments. In addition, Pan‐Kla level, SP1 lactylation and the proportion of M1 macrophages were also upregulated in CSE‐treated MH‐S cells (Figure S2G–I). To verify whether lactylation occurs at the K694 site of SP1, the lysine (K) at position 694 was replaced with arginine (R) to mimic delactylation, and MH‐S cells were transduced using a lentiviral vector. IP analysis showed that compared with SP1‐Wild Type(WT), lactylation was markedly reduced in SP1‐K694R (Figure 3G), indicating that K694 lactylation may be a key component of SP1 lactylation. Notably, conservation of SP1‐K694 and its neighbouring residues was observed across *Homo sapiens*, *Mus musculus*, and *Rattus norvegicus*, based on UniProt (https://www.uniprot.org/uniprotkb) and CPLM (http://cplm.biocuckoo.cn/index.php) (Figure S2D). Therefore, SP1‐K694 was selected as the main target in this study. In addition, the SP1‐K694R mutation in MH‐S cells reduced the proportion of M1 macrophages (Figure 3H,I) and the levels of IL‐1β, IL‐6, and TNF‐α in the cell supernatant compared with the WT‐SP1 group under CSE treatment (Figure 3J).

To mimic constitutive lactylation, an SP1‐K694Q mutant was generated by replacing lysine (K) at position 694 with glutamine (Q). The mutant construct was transduced into MH‐S cells via a lentiviral vector. IP analysis showed that, compared with the WT‐SP1 group, lactylation was increased in SP1‐K694Q (Figure S3A). Under CSE treatment, both flow cytometry and IF staining revealed a higher proportion of M1 macrophages in the SP1‐K694Q group than in theSP1‐WT group (Figure S3B,C), along with higher levels of IL‐1β, IL‐6, and TNF‐α in the cell supernatant (Figure S3D).

Furthermore, we generated a site‐specific lactylation antibody targeting SP1 K694 (SP1‐K694la) (Figure S3E). Dot blot analysis revealed that the antibodies specifically recognized the lactylated peptides, with no detectable binding to the unmodified peptides at the corresponding sites (Figure S3F). In addition, the R mutation resulted in a reduction in SP1‐K694la following IP (Figure S3G). The expression of SP1‐K694la was increased in CSE‐induced macrophages compared with the control group. Under CSE treatment, the SP1‐K694R mutation decreased SP1‐K694la expression detected by Western blotting (Figure S3E).

### 2.4 SP1 K694 Lactylation Increased M1 Macrophage Polarization in Mice With COPD

**FIGURE 4:**
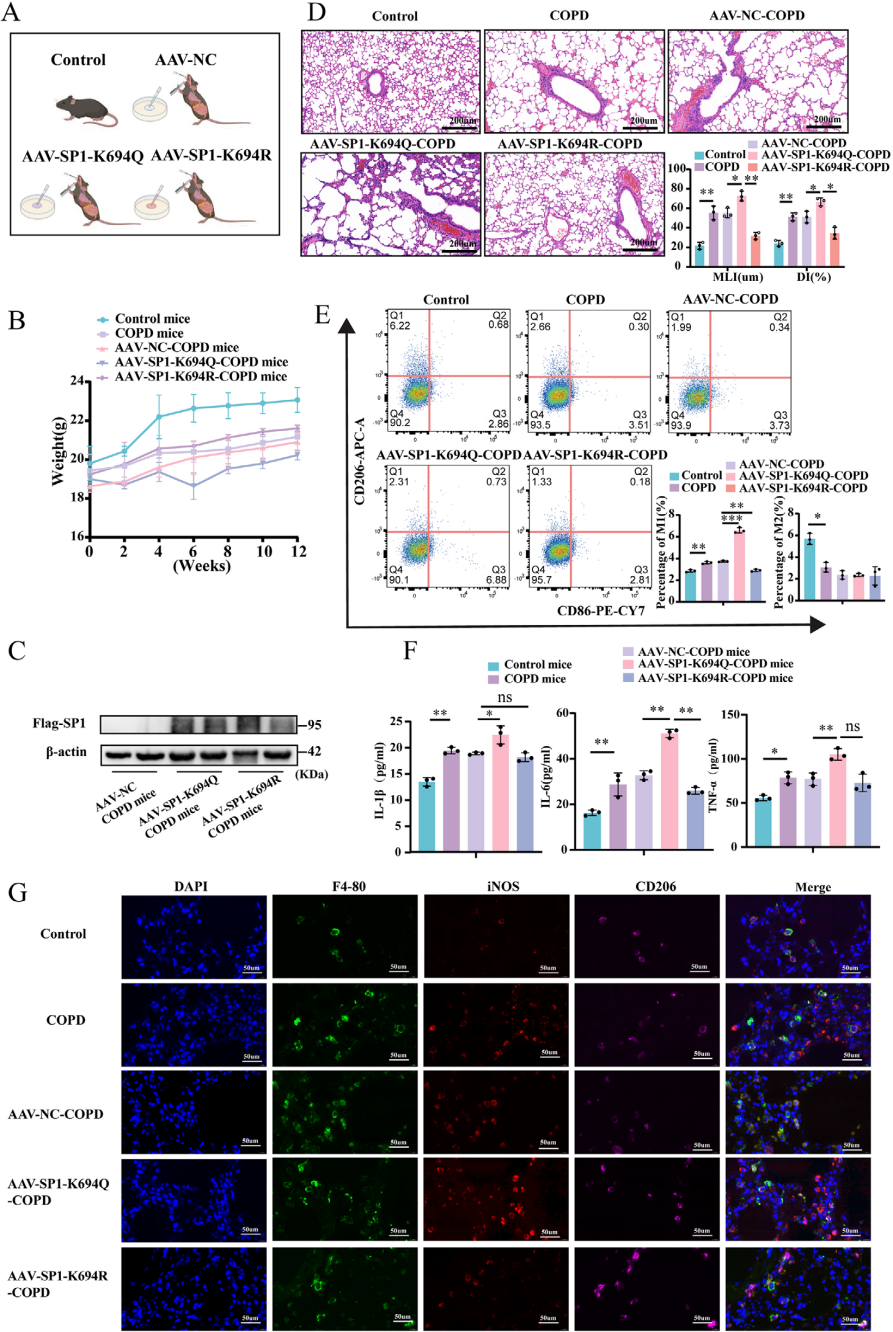
SP1‐K694 lactylation increased M1 macrophage polarization in COPD mice. (A) Schematic of the experimental design. AAV‐SP1‐K694R, AAV‐SP1‐K694Q, and AAV‐NC were administered via intratracheal injection at the end of the second week of cigarette smoke exposure. (B) Body weight changes in mice across different groups. (C) The FLAG expression in COPD model mice intratracheally injected with AAV‐NC, AAV‐SP1‐K694Q and AAV‐SP1‐K694R. (D) H&E staining of mouse lung tissue (scale bar = 200 µm). (E) CD86 and CD206 expression in lung macrophages detected by flow cytometry, and (F) Concentrations of IL‐1β, IL‐6, and TNF‐α in BALF from different groups. (G) iNOS and CD206 expression detected by IF. Representative images show lung sections co‐stained with anti‐F4/80 (green, macrophage marker), anti‐iNOS (red), or anti‐CD206 (pink) in different groups (scale bar = 100 µm). *n* = 8 mice per group; COPD, chronic obstructive pulmonary disease; BALF, bronchoalveolar lavage fluid; DAPI, 4′,6‐diamidino‐2′‐phenylindole; DI, destructive index; MLI, mean linear intercept. ANOVA+Tukey were used,^*^ *p* < 0.05,^**^ *p* < 0.01, ^***^ *p* < 0.001.

We generated SP1‐K694R (delactylation‐mimicking) and SP1‐K694Q (constitutive lactylation‐mimicking) mutations, delivered them to mice via intratracheal instillation using AAV after the second week of cigarette smoke exposure, and continued smoke exposure for a total of 12 weeks (Figure 4A). Successful viral transduction was confirmed by FLAG protein expression (Figure 4C). It was observed that the emphysema and airway inflammation cell infiltration detected by Hematoxylin and eosin(H&E staining, the proportion of M1 macrophage, as well as the concentrations of IL‐1β, IL‐6, and TNF‐αin BALF, were similar between Control mice and AAV‐NC mice (Figure S5B–F). Body weight and lung function were improved, and emphysema and airway inflammation were alleviated in mice treated with adeno‐associated virus (AAV)‐SP1‐K694R compared with those in the COPD group (Figure S4A and Figure 4B,D). In addition, a lower proportion of M1 macrophages in lung tissue and reduced levels of IL‐6 in BALF were observed in COPD mice treated with AAV‐SP1‐K694R (Figure 4E–G).

By contrast, in mice with COPD administered AAV‐SP1‐K694Q, body weight was reduced, while lung function was impaired and emphysema and airway inflammation cell infiltration(H&E staining) were aggravated. Moreover, the proportion of M1 macrophages in lung tissue and the levels of IL‐1β, IL‐6, and TNF‐α were increased (Figure S4A and Figure 4B–G).

### 2.5 SP1‐K694 Lactylation Induced CSE‐Induced M1 Macrophage Polarization by Regulating TREM1 Expression

**FIGURE 5:**
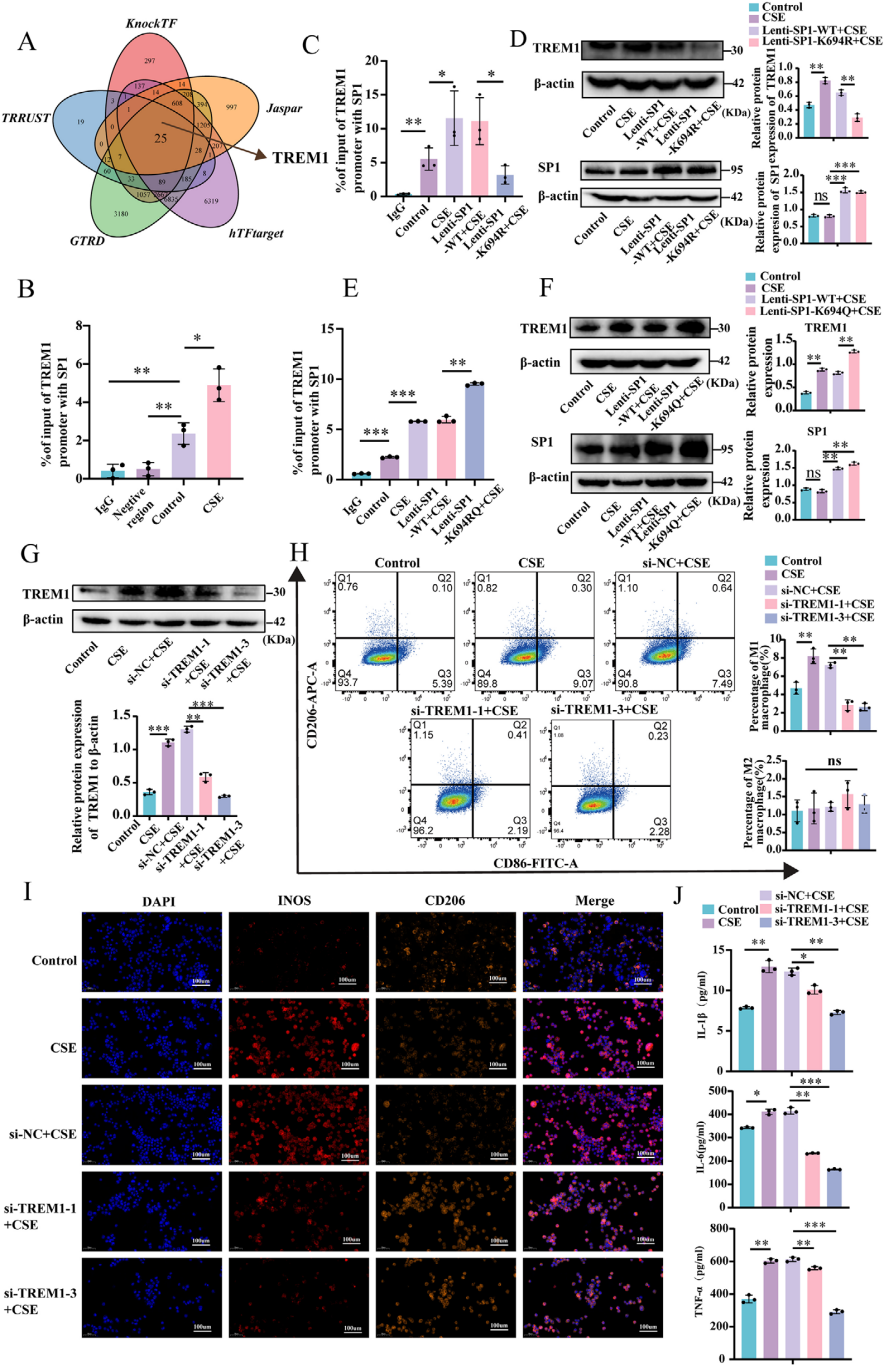
SP1‐K694 lactylation induced M1 macrophage polarization by regulating TREM1 expression in CSE‐induced MH‐S cells. (A) Venn diagram showing overlapping potential SP1 target genes identified across six transcription factor databases. ChIP‐qPCR analysis of the TREM1 promoter using an anti‐SP1 antibody in (B) CSE‐induced MH‐S cells and (C) CSE‐induced MH‐S cells expressing the SP1‐K694R mutant. (D) Expression of SP1 and TREM1 in CSE‐induced MH‐S cells following overexpression of SP1‐WT or the SP1‐K694R mutant. (E) ChIP‐qPCR analysis of the TREM1 promoter using an anti‐SP1 antibody and (F) expression of SP1 and TREM1 in CSE‐induced MH‐S cells expressing the SP1‐K694Q mutant. (G)TREM1 protein expression, (H) CD86 and CD206 expression detected by flow cytometry, (I) iNOS (red) and CD206 (orange) expression detected by IF (scale bar = 100 µm), and (J) concentrations of IL‐1β, IL‐6, and TNF‐α in the cell supernatant of CSE‐induced MH‐S cells transfected with si‐TREM1.ANOVA+Tukey were used,^*^ *p* < 0.05, ^**^ *p* < 0.01, ^***^ *p* < 0.001.

The SP1‐K694 residue is located within the C2H2 zinc finger domain of SP1. Using six transcription factor databases, we identified 25 overlapping potential target genes of SP1; among these, TREM1 was selected because of its association with COPD and macrophage polarization (Figure 5A). Three predicted SP1 binding sites were identified in the TREM1 promoter using the JASPAR database, and ChIP‐qPCR showed that SP1 binds to the region 1794 to 1803 bp upstream of the transcription start site of TREM1 (Figure S4B,C). As shown in Figure 5B, ChIP‐qPCR revealed that the enrichment of SP1 at the negative control region was comparable to that of the IgG control, with no statistical difference observed between these two groups. However, the enrichment at the putative SP1‐binding site (positive region) was markedly elevated compared to both the negative control and IgG groups. The results also showed that enrichment of SP1 at the TREM1 promoter was further enhanced following CSE treatment (Figure 5B). TREM1 protein expression was increased in CSE‐treated MH‐S cells (Figure S4D). Meanwhile, the K694R mutation impaired SP1 enrichment at the TREM1 promoter, and TREM1 protein expression was also decreased following the K694R mutation (Figure 5C,D), indicating that this process is regulated by SP1 lactylation. Furthermore, the K694Q mutation promoted SP1 binding to the TREM1 promoter and concomitantly increased TREM1 protein expression (Figure 5E,F). To determine whether SP1‐K694 lactylation directly affects DNA‐binding affinity, EMSA was performed using recombinant SP1‐WT, SP1‐K694Q (lactylation‐mimetic), and SP1‐K694R (lactylation‐deficient) proteins with a biotin‐labelled probe spanning the SP1‐binding motif within the TREM1 promoter. As shown in Figure S4E, the SP1‐K694Q mutant exhibited significantly enhanced DNA‐binding affinity compared to SP1‐WT, whereas the SP1‐K694R mutant showed markedly reduced binding. These results demonstrate that lactylation at K694 enhances DNA‐binding affinity of SP1 to the TREM1 promoter.

To directly validate the role of TREM1 in macrophage polarization, MH‐S cells were transfected with TREM1 siRNA (Figure S7A,B). Under CSE treatment, TREM1 knockdown reduced TREM1 expression (Figure 5G), decreased M1 macrophage polarization, and lowered levels of IL‐1β, IL‐6, and TNF‐α (Figure 5H–J).

**FIGURE 6:**
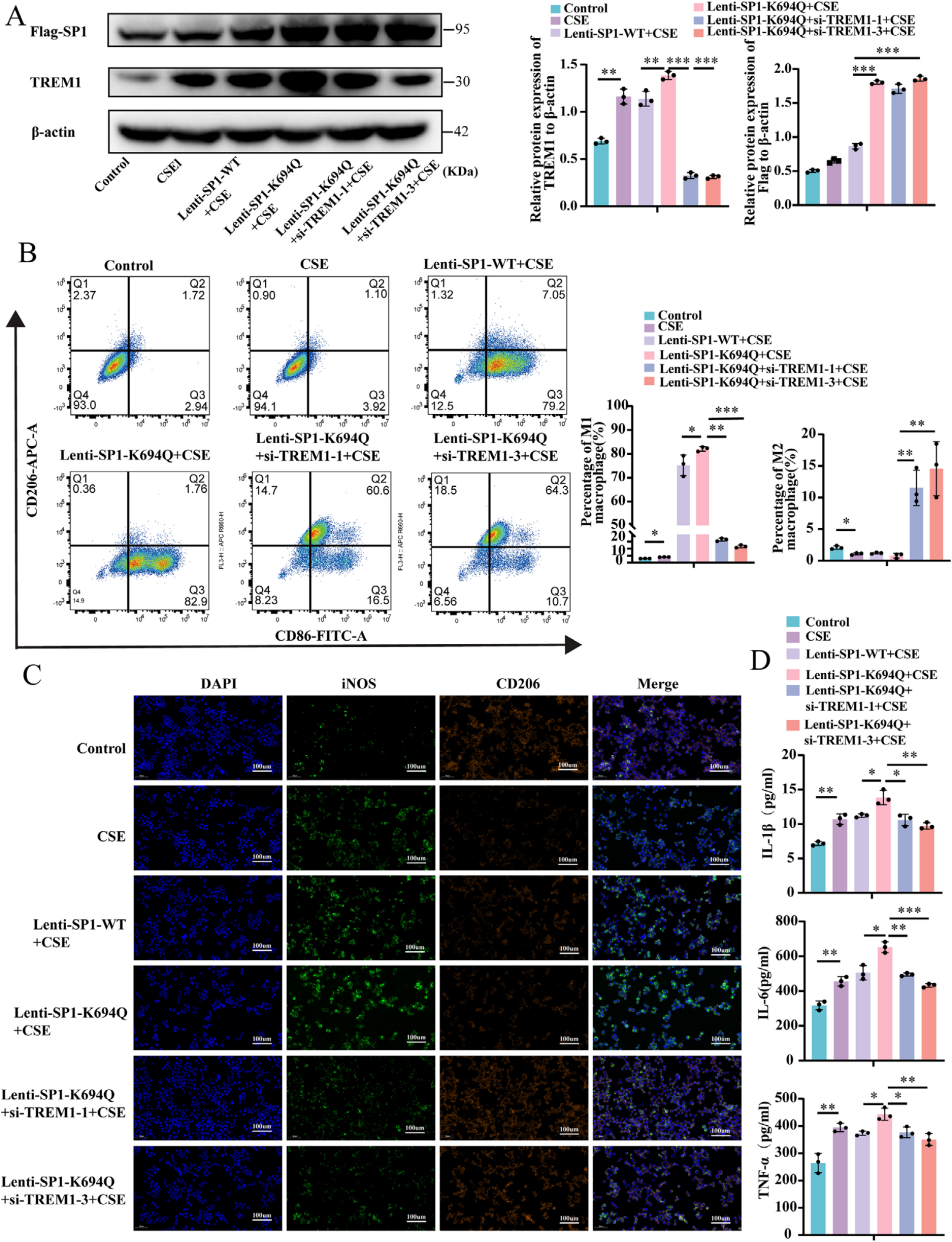
TREM1 knockdown alleviates SP1‐K694Q‐mediated increase in M1 macrophage polarization in CSE‐treated MH‐S cells. (A) Protein expression of Flag‐SP1 and TREM1 in CSE‐induced MH‐S cells transfected with si‐TREM1 and overexpressing the SP1‐K694Q mutant via lentiviral transduction. (B) CD86 and CD206 expression detected by flow cytometry, (C) iNOS (green) and CD206 (orange) expression detected by IF (scale bar = 100 µm), and (D) concentrations of IL‐1β, IL‐6, and TNF‐α in the cell supernatant in CSE‐induced MH‐S cells transfected with si‐TREM1 and overexpressing the SP1‐K694Q mutant.

Furthermore, MH‐S cells expressing the K694Q mutation were transfected with si‐TREM1 (Figure 6A). As expected, TREM1 knockdown alleviated SP1‐K694Q ‐mediated increasing in M1 macrophage polarization and the secretion of IL‐1β, IL‐6, and TNF‐α (Figure 6B–D).

Finally, MH‐S cells expressing the K694R mutation were transfected with Overexpression (OE)‐TREM1 (Figure S6A). Notably, overexpression of TREM1 reversed the SP1‐K604R mutation‐induced decline in the M1 polarization ratio and the levels of IL‐1β, IL‐6, and TNF‐α (Figure S6B–D).

### 2.6 SP1‐K694 Lactylation Induced M1 Macrophage Polarization by Regulating TREM1 Expression in Mice With COPD

**FIGURE 7:**
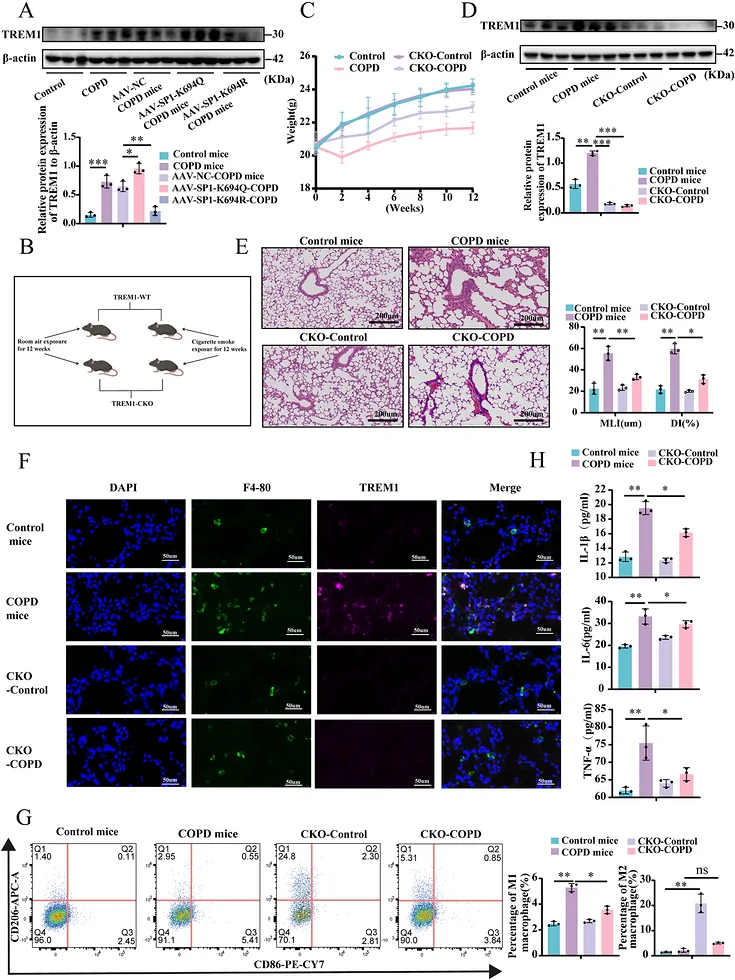
SP1‐K694 lactylation induced M1 macrophage polarization by regulating TREM1 expression in COPD mice. (A) TREM1 protein expression in the lungs of COPD mice following intratracheal administration of AAV‐SP1‐K694Q and AAV‐SP1‐K694R. (B) Schematic of the experimental design. WT and TREM1‐CKO mice were exposed to fresh air or cigarette smoke for 12 weeks. (C–H) Effects of macrophage‐specific TREM1 knockout on emphysema, airway inflammation, and macrophage polarization in COPD mouse lung tissue. (C) Body weight changes, (D) TREM1 protein expression, (E) H and E staining of lung tissue, and (F) IF staining of TREM1 in lung macrophages. Representative images show lung sections co‐stained with anti‐F4/80 (green, macrophage marker) and anti‐TREM1 (red); nuclei were counterstained with DAPI (blue). (G) CD86 and CD206 expression in lung macrophages detected by flow cytometry, and (H) Concentrations of IL‐1β, IL‐6, and TNF‐α in BALF from different groups. *n* = 8 mice per group; COPD, chronic obstructive pulmonary disease; BALF, bronchoalveolar lavage fluid; DAPI, 4′,6‐diamidino‐2′‐phenylindole; DI, destructive index; MLI, mean linear intercept. ANOVA+Tukey were used,^*^ *p* < 0.05, ^**^ *p* < 0.01, ^***^ *p* < 0.001.

TREM1 protein expression was elevated in mice with COPD compared with control mice (Figure S4F). We further assessed TREM1 expression in lung macrophages of mice with COPD administered AAV‐SP1‐K694R or AAV‐SP1‐K694Q via IF and western blot. The results showed that the K694R mutation reduced TREM1 expression, whereas the K694Q mutation increased TREM1 expression (Figure 7A and Figure S5A).

Furthermore, we established macrophage‐specific TREM1 knockout (CKO) mice and used their littermate WT mice as controls (Figure 7B). TREM1 deletion in macrophages was confirmed at the DNA, RNA, and protein levels (Figure S7C–E). Compared with WT‐COPD mice, CKO‐COPD mice exhibited increased body weight, improved lung function, and reduced emphysema and airway inflammation cell infiltration, as assessed by H&E staining (Figure 7C,E and Figure S7F). Both IF staining and western blot showed that TREM1 expression was significantly decreased in CKO‐Control and CKO‐COPD mice (Figure 7D,F). In addition, CKO‐COPD mice showed a reduced proportion of M1 macrophages in the lung and lower concentrations of IL‐1β, IL‐6, and TNF‐α in BALF (Figure 7G,H and Figure S7G).

**FIGURE 8:**
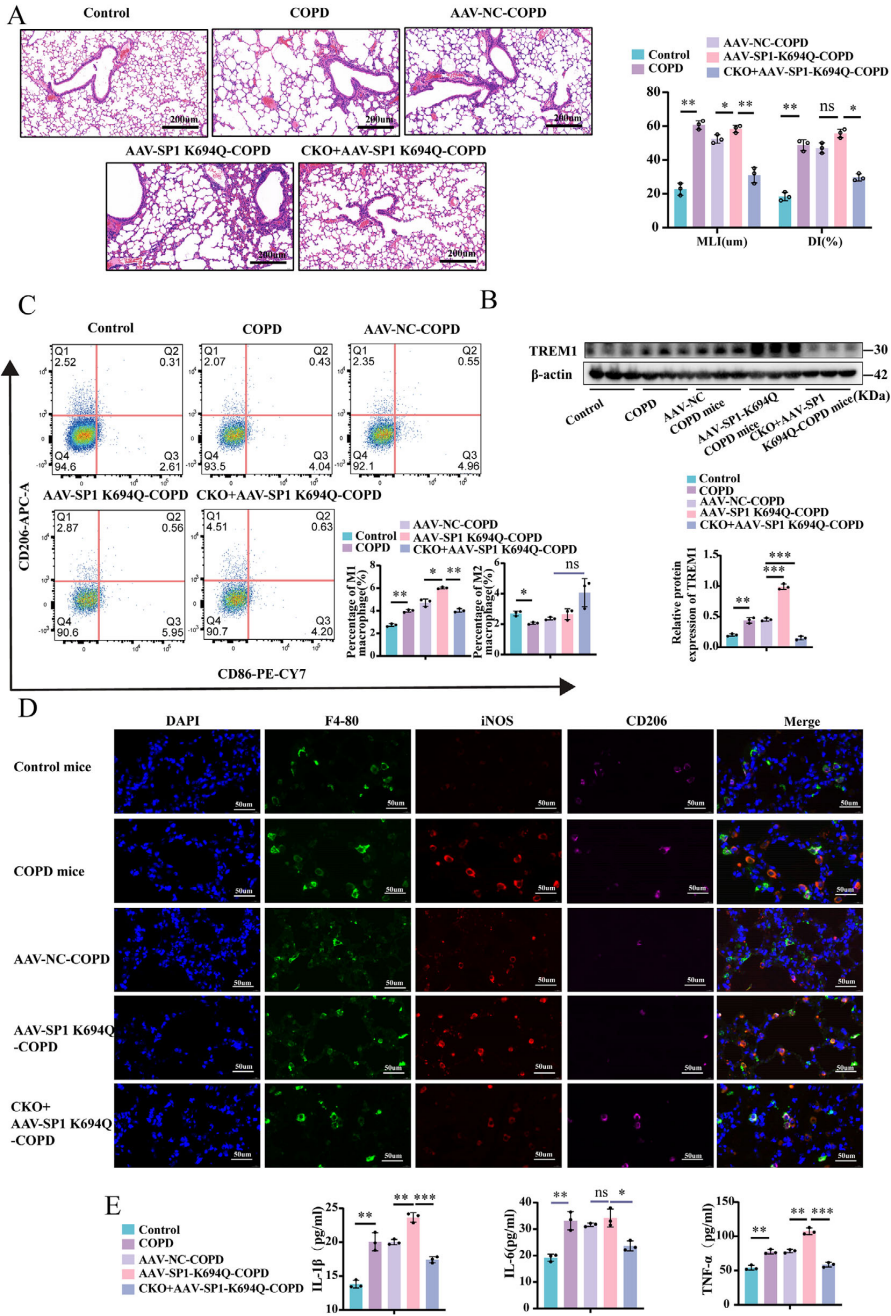
TREM1‐CKO alleviates SP1‐K694 hyperlactylation‐mediated M1 macrophage in COPD mice. A‐E. The emphysema and airway inflammation, as well as macrophagepolarization, in COPD model mice administered with TREM1‐CKO and SP1‐K694Q mutation at the same time. The HE staining of mouse lung tissue (A), the protein expression of TREM1 of mice lung tissue (B), CD86 and CD206 expression in macrophages of mouse lung tissue detected by Flow cytometry(C), and IF staining of iNOS and CD206 expression in lung macrophages. Representative images show lung sections co‐stained with anti‐F4/80 (green), anti‐iNOS (red), or anti‐CD206 (pink) (scale bar = 100 µm) (D), Concentrations of IL‐1β, IL‐6, and TNF‐α in BALF (E) from different groups. *n* = 8 mice per group; COPD, Chronic obstructive pulmonary disease; BALF:Bronchoalveolar Lavage Fluid; DAPI: 4’,6‐diamidino‐2’‐phenylindole; DI: Destructive index; MLI: Mean linear intercept; ANOVA+Tukey were used, ^*^ *p* < 0.05; ^**^ *p* < 0.01; ^***^ *p* < 0.001.

Additionally, WT and CKO mice were treated with AAV‐SP1‐K694Q via intratracheal instillation (AAV‐SP1‐K694Q‐COPD and CKO+AAV‐SP1‐K694Q‐COPD groups, respectively). While AAV‐SP1‐K694Q‐COPD mice exhibited increased M1 macrophage polarization and exacerbated airway inflammation cell infiltration, inhibition of TREM1 in CKO+AAV‐SP1‐K694Q‐COPD mice attenuated emphysema and airway inflammation, as assessed by H&E staining (Figure 8A,B and Figure S8). Moreover, CKO+AAV‐SP1‐K694Q‐COPD mice showed a reduced proportion of M1 macrophages in the lung and lower concentrations of IL‐1β, IL‐6, and TNF‐α in BALF (Figure 8C–E).

### 2.7 Identification of ACAT1 as an SP1 K694 Lactyltransferase

**FIGURE 9:**
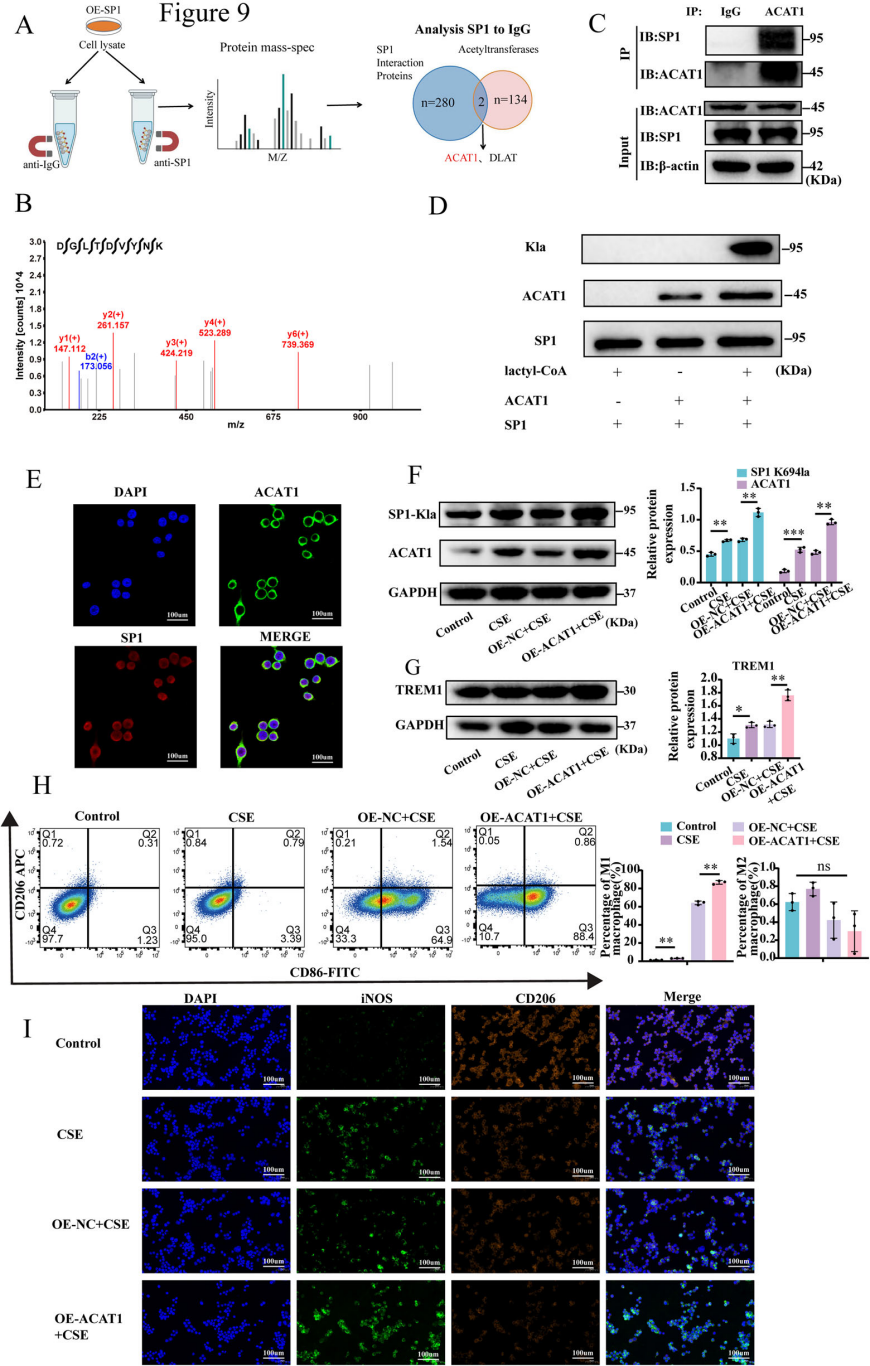
Identification of ACAT1 as an SP1‐K694 lactyltransferase. (A)Schematic diagram illustrating the identification of SP1‐interacting acetyltransferases using IP combined with MS. (B) IP‐MS analysis identifying ACAT1 as an SP1‐interacting protein with acyltransferase activity. (C) Co‐immunoprecipitation analysis confirming the interaction between ACAT1 and SP1. (D) In vitro ACAT1 lactylation assay. Purified recombinant SP1 was incubated with recombinant ACAT1 with or without lactyl‐ CoA. Kla levels of SP1 were analyzed by WB. (E) Colocalization of SP1 and ACAT1 in MH‐S cells, which were co‐stained with anti‐ACAT1 (green) and anti‐SP1 (red). Nuclei were counterstained with DAPI (blue) (scale bar = 100 µm). Protein expression of (F) ACAT1 and SP1‐K694la, and (G) TREM1, in CSE‐induced MH‐S cells following ACAT1 overexpression. (H) CD86 and CD206 expression detected by flow cytometry, and (I) iNOS (green) and CD206 (orange) expression detected by IF (scale bar = 100 µm) in CSE‐induced MH‐S cells following ACAT1 overexpression. ANOVA+Tukey were used, ^*^ *p* < 0.05, ^**^ *p* < 0.01.

To identify regulators of SP1 lactylation, we conducted immunoaffinity purification followed by Liquid Chromatography‐Mass Spectrometry (LC‐MS/MS in SP1‐overexpressing MH‐S cells and identified two potential interacting acetyltransferases, acetyl‐CoA acetyltransferase 1 (ACAT1) and dihydrolipoamide S‐acetyltransferase (DLAT) (Figure 9A). Following overexpression of ACAT1 and DLAT in MH‐S cells, SP1‐K694la levels were markedly increased only in ACAT1‐overexpressing cells, indicating that ACAT1, but not DLAT, possesses lactyltransferase activity towards SP1 (Figure S9A‐B). Following the identification of ACAT1 as a potential acyltransferase responsible for SP1 lactylation (Figure 9B), we used HDOCK (http://hdock.phys.hust.edu.cn/) to model putative the three‐dimensional interaction between SP1 and ACAT1 (Figure S9D). We further validated the interaction between ACAT1 and SP1 in MH‐S cells by immunoprecipitation (Figure 9C). IF staining also showed colocalization of ACAT1 and SP1 in MH‐S cells (Figure 9E). To assess whether ACAT1 mediates direct lactyl group transfer from lactyl‐CoA to SP1, we set up an in vitro lactylation system. After incubating purified SP1 and ACAT1 with or without lactyl‐CoA, we found that obvious SP1 lactylation occurred only in the presence of ACAT1 and lactyl‐CoA (Figure 9D). Pan‐Kac levels remained unchanged upon ACAT1 overexpression (Figure S9C). We found that ACAT1 expression was increased in CSE‐induced MH‐S cells and in the lungs of mice with COPD (Figure S9E,F). Furthermore, under the overexpression of K694Q background, TREM1 CKO in mice or si‐TREM1 in MH‐S cells did not elevate ACAT1 protein levels (Figure S9G,H). Upon CSE treatment in MH‐S cells, ACAT1 overexpression enhanced the expression of SP1‐K694la and TREM1 (Figure 9F,G). Meanwhile, both flow cytometry and IF staining showed that the proportion of M1 macrophages was increased (Figure 9H,I). Moreover, knockdown of ACAT1 reduced the expression of SP1‐K694la and TREM1 (Figure S10A,B) and attenuated M1 macrophage polarization in CSE‐treated MH‐S cells (Figure S10C,D).

### 2.8 Lactylation Level and Expression of TREM1 in Patients With COPD

**FIGURE 10:**
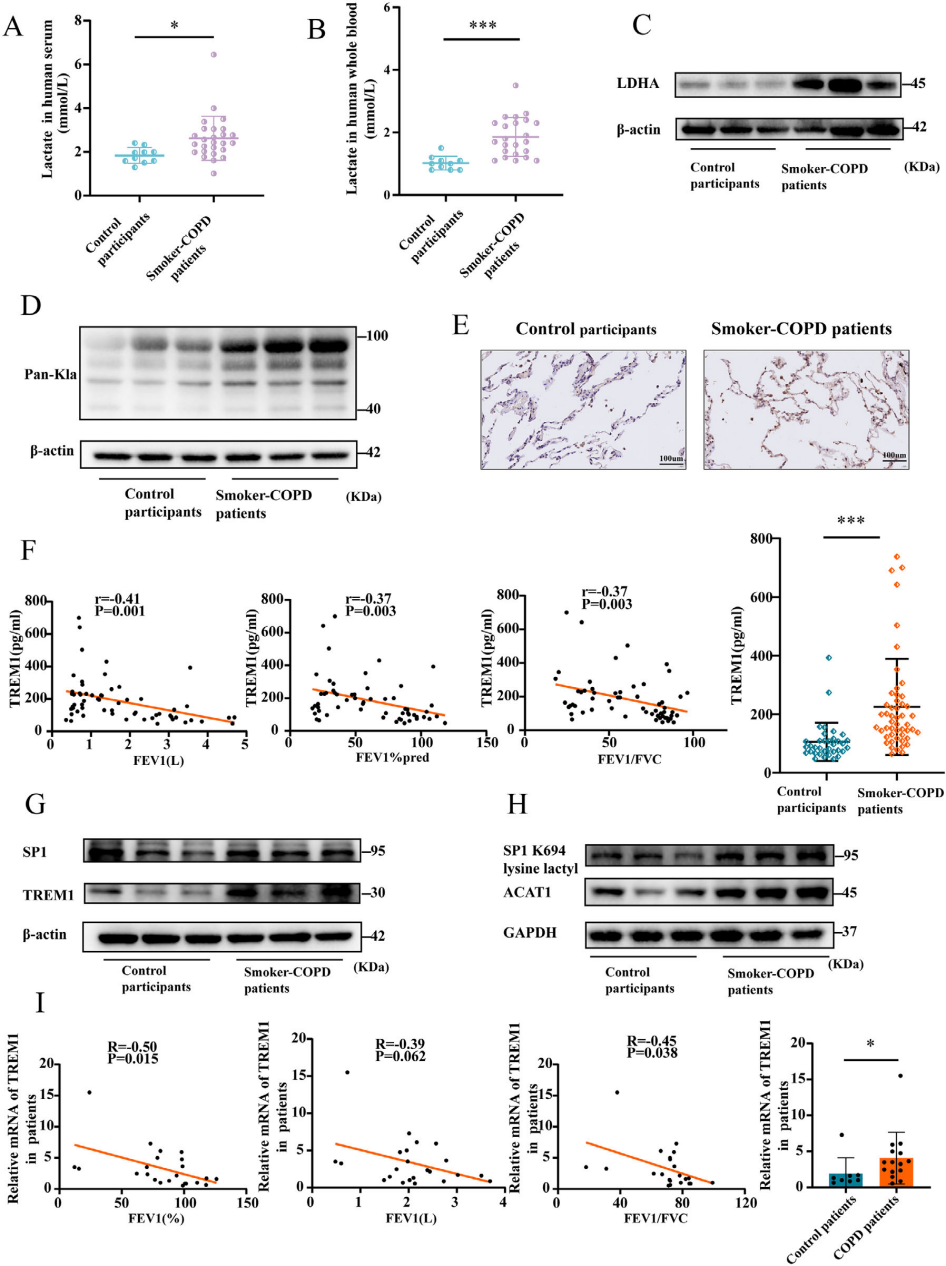
Lactylation levels and expression of TREM1 and ACAT1 in patients with COPD. Lactate in human serum (A) and lactate in human whole blood(B) were detected in control participants and COPD patients; Control participants (*n* = 10) and COPD patients (*n* = 23). C‐E. The expression of LDHA and Pan‐Kla was assessed by Western blot, and Pan‐Kla were assessed by IHC(scale bar = 100 µm) in control participants and COPD patient's lung tissue; Control participants (*n* = 15) and COPD patients (*n* = 13). (F)The concentration of TREM1 in plasma was detected by ELISA assay in control participants and COPD patients, and the correlation between the concentration of TREM1 and FEV1, FEV1% or FEV1/FVC; Control participants (*n* = 10) and COPD patients (*n* = 23). The expression of SP1 and TREM1(G), SP1 K694la and ACAT1(H) in control participants and COPD patient's lung tissue; Control participants (*n* = 15) and COPD patients (*n* = 13). (I) The mRNA expression of TREM1 in control participants and COPD patient's lung tissue and the correlation between the concentration of TREM1 and FEV1, FEV1% or FEV1/FVC; Control participants (*n* = 15) and COPD patients (*n* = 13).*t*‐test for analysis was used, ^*^ *p*<0.05; ^***^ *p*<0.001.

We collected peripheral blood from control participants and patients with COPD, and the demographic data are shown in Table S1. Lactate levels in plasma and blood were increased in patients with COPD (Figure 10A,B). Lactate levels were positively correlated with smoking index, after adjusted with age, sex, Body Mass Index (BMI) and GOLD grade. Compared with GOLD 1 grade, the lactate levels in GOLD 3 and 4 groups were higher (Table S3 and Figure S11A,B). In addition, the concentration of TREM1 in plasma was significantly elevated in patients with COPD and was negatively correlated with FEV1, FEV1%pred, and FEV1/FVC (Figure 10F). Following adjustment for age, sex, BMI, and smoking index, TREM1 concentration showed a positive correlation with smoking index; notably, it was significantly higher in patients with GOLD grades 3 and 4 than in those with grade 1 (Figure S11C,D and Table S4).

Furthermore, lung tissue samples were collected from control participants and patients with COPD, and the clinical characteristics of these participants are shown in Table S2. Both IHC and Western blot results showed that Pan‐Kla levels were increased in patients with COPD (Figure 10D,E). The protein expression of LDHA, TREM1, SP1‐K694la, and ACAT1 was also elevated in COPD (Figure 10C,G,H). In addition, TREM1 mRNA expression in lung tissue was increased in patients with COPD and was negatively correlated with FEV1, FEV1%pred, and FEV1/FVC (Figure 10I). After adjustment for age, sex, BMI and smoking index in the multivariable linear model, the association between TREM1 and FEV1, FEV1% or FEV1/FVC remained robust (Table S5). IF analysis showed colocalization of CD68 (a macrophage marker) and TREM1, and indicated that TREM1 expression in macrophages was increased in patients with COPD (Figure S10E). In addition, IF analysis showed that M1 macrophages were increased in patients with COPD (Figure S10F,G).

## 3 Discussion

The main finding of this study is that SP1‐K694 lactylation enhances TREM1 transcription and thereby regulates M1 macrophage polarization, contributing to airway inflammation in COPD. Furthermore, this study identifies ACAT1 as a lactyltransferase involved in SP1‐K694 lactylation. Airway inflammation is one of the main pathogenic mechanisms of COPD, leading to airway remodelling, increased mucus secretion, alveolar wall destruction, and small airway narrowing [25]. Long‐term inhalation of harmful particles or gases (such as cigarette smoke) can damage airway epithelial cells, activating innate and adaptive immune responses and leading to extensive infiltration of inflammatory cells, including neutrophils, macrophages, and lymphocytes, into the airway wall. These cells release various inflammatory mediators, thereby establishing a chronic and persistent inflammatory state [26, 27]. Macrophage polarization imbalance is also a key contributor to airway inflammation. Previous studies have reported inconsistent findings regarding macrophage phenotypes in COPD, with some showing an increase in M1 macrophages that secrete proinflammatory cytokines [9], while others have reported an increase in M2 macrophages associated with airway remodelling [28]. In the present study, the proportion of M1 macrophages was elevated in CSE‐induced macrophages and in the lungs of COPD mice exposed to cigarette smoke for 12 weeks. Macrophage phenotypes in COPD are associated with disease subtypes. Our model recapitulates the early pathogenic process of COPD rather than the late stage of the disease; therefore, the phenotype is predominantly characterized by an increase in M1 macrophages.

Lactylation, a protein post‐translational modification, is implicated in diverse cellular processes and disease pathogenesis [29, 30]. Lactylation plays an important role in regulating macrophage polarization, mediating both the production of inflammatory mediators and tissue repair capacity. Histone lactylation exhibits promoter‐specific enrichment at M2‐associated loci (e.g., Arg1, VEGFα), thereby facilitating transcriptional activation of anti‐inflammatory programmes [12, 15, 31]. However, the mechanistic interplay between lactylation and proinflammatory M1 macrophage polarization remains to be fully elucidated. Our data demonstrate that lactate levels are upregulated in CSE‐treated macrophages and that subsequent glycolytic suppression using the LDHA inhibitor ， oxamate ， attenuates M1 macrophage polarization. These findings are consistent with previous reports showing that lactate drives M1 macrophage polarization [16] and that LDHA is associated with lactylation in COPD [17].

We identified lactylation residues using whole‐protein lactylation modification‐omics analysis and found that SP1‐K694 lactylation was increased in CSE‐induced macrophages. This finding was also confirmed in the lungs of mice with COPD and in patients with COPD using a site‐specific antibody (SP1‐K694la). Furthermore, both in vitro and in vivo experiments demonstrated that inhibition of SP1‐K694 lactylation decreased M1 macrophage polarization, whereas the K694Q mutation to mimic SP1‐K694 lactylation enhanced M1 macrophage polarization. These findings identify SP1‐K694 lactylation as an epigenetic signature of M1 macrophage polarization and pro‐inflammatory activation. This observation is consistent with findings in pulmonary fibrosis, where the H3K18la/NOS2 axis drives M1 macrophage polarization in the fibrotic microenvironment [16]. SP1 has been reported to mediate endothelial progenitor cell senescence in COPD [32]; however, its role in macrophages has not been previously explored. Our study provides the first evidence, to our knowledge, that non‐histone lactylation plays a functional role in COPD pathogenesis. This distinguishes our work from recent reports that focused exclusively on histone lactylation, which has been investigated in COPD models induced by polycyclic aromatic hydrocarbons, such as benzo[a]pyrene and 1‐nitropyrene, with studies focusing on the mechanisms underlying histone modification and lung epithelial cell senescence [17, 18]. Yang et al. identified the H4K12la‐CD38‐NAD^+^axis in AEC2 senescence [33]. By contrast, our study explores lactylation mechanisms in a cigarette smoke‐induced COPD model and highlights the role of SP1 lactylation, a non‐histone modification, in macrophage polarization. This non‐histone lactylation mechanism highlights the multifaceted nature of lactylation regulation in COPD.

Although SP1‐K694 lactylation is involved in macrophage polarization, the specific mechanism by which this modification drives M1 polarization remains unclear. As a key transcription factor, SP1 is predicted to regulate TREM1, a molecule associated with macrophage polarization [34]. ChIP‐qPCR identified TREM1 as a direct transcriptional target of SP1‐K694 lactylation. Notably, only one of three JASPAR‐predicted SP1‐binding sites in the TREM1 promoter was validated by ChIP‐qPCR. This highlights that actual transcription factor occupancy is governed by chromatin state and cooperative interactions, and it is common for only one or two sites to be the primary regulators of transcription [24, 35]. Our validated the site (region 1794 to 1803 bp) therefore represents the primary functional SP1‐binding site for TREM1 transcription in macrophages. CSE exposure induced TREM1 upregulation and increased SP1 enrichment at its promoter. Reducing SP1‐K694 lactylation (K694R mutation) decreased TREM1 expression and SP1 enrichment, whereas mimicking lactylation (K694Q mutation) increased both. This finding supports the role of TREM1 as a marker and mediator of M1 macrophage polarization. Previous studies have shown that histone lactylation can stimulate gene transcription on chromatin [36]. In hypoxic microglia, the non‐histone transcription factor Yin Yang 1 (YY1) exhibits increased lactylation, which enhances its binding to the FGF2 promoter and upregulates FGF2 expression [37]. Together, these findings suggest a mechanism by which lactylation facilitates chromatin accessibility and transcriptional activation. Our EMSA data demonstrate that SP1‐K694 lactylation directly enhances the DNA‐binding affinity of SP1 to the TREM1 promoter. This is consistent with studies showing that lactylation of other transcription factors, including RelA [38] and p53^19^, directly modulates their DNA‐binding activities. Whereas we cannot exclude the possibility that lactylation also promotes the recruitment of transcriptional co‐activators to further stabilize SP1 occupancy. Future studies using Co‐IP assays will be required to determine whether SP1‐K694 lactylation alters its interaction with p300, WDR5, or other coactivators.

TREM1 is a transmembrane glycoprotein primarily expressed in monocytes, macrophages, and neutrophils, and it promotes the release of proinflammatory mediators and amplification of inflammatory responses [39]. Our study showed that TREM1 expression was increased in patients with COPD, mice with COPD, and CSE‐induced macrophages, and that TREM1 knockdown reduced M1 macrophage polarization both in vivo and in vitro. Previous studies have demonstrated that TREM1 promotes M1 macrophage polarization through multiple signalling pathways, including STAT3/HIF‐1α [40] and TLR4/NF‐κB [41], thereby enhancing the release of proinflammatory mediators. However, the precise mechanism by which TREM1 drives M1 polarization was not explored in the present study. There may exist additional SP1 targets beyond TREM1 that are likely involved in the SP1‐K694la, which may drive M1 polarization. For the additional candidate genes identified by prediction, future ChIP‐qPCR and functional studies will be required to establish their roles in the SP1‐K694la‐driven M1 polarization. Nevertheless, our findings establish TREM1 as a critical downstream target gene of SP1‐K694la in the regulation of M1 polarization.

In the field of protein acylation, nearly all identified short‐chain acylation modifications have been shown to be regulated by acyltransferases and deacylases [42]. Through IP‐MS analysis, we identified ACAT1 as a potential acyltransferase involved in SP1 lactylation, which interacts with SP1. IP analysis further confirmed a direct interaction between ACAT1 and SP1. We acknowledge that the HDOCK‐predicted ACAT1‐SP1 interface has not been validated by mutagenesis. This modelling served as supportive evidence for the interaction already confirmed by LC‐MS/MS and Co‐IP, rather than to define the precise interface. Systematic mutagenesis of the predicted interface remains an important direction for future investigation. We also found that ACAT1 overexpression enhanced the expression of SP1‐K694la and TREM1, whereas inhibition of ACAT1 reduced the expression of SP1‐K694la and TREM1 in macrophages. These results suggest that ACAT1 may function as a lactyltransferase mediating SP1‐K694 lactylation in CSE‐induced macrophages. ACAT1 is a key regulatory enzyme which functions as an acetyltransferase, mediating the acetylation of multiple proteins [43]. Notably, a recent study reported that ACAT1 regulates IDH2 lactylation to promote angiogenesis in diabetic myocardial infarction in mice [44].

### 3.1 Limitations and Future Perspectives

First, we acknowledge that the lysine‐to‐glutamine (K694Q) substitution, while have been used as a functional mimic for lysine acylation modifications including acetylation and lactylation(Kla). However, glutamine lacks the steric bulk and hydrogen‐bonding capacity conferred by the hydroxyl group of the lactyl moiety. Further studies need to perform targeted mass spectrometry to directly confirm K694 lactylation in the K694Q mutant. Although genetic code expansion (GCE) for site‐specific incorporation of lactyl‐lysine would be the gold standard for studies, as the site‐specific lactylation system is a transient expression system, for our experiments with a longer duration, we mimicked lysine lactylation using Q to substitute K. Besides, Ma, C et al. [45] indicated that functional trends of lysine lactylation simulated by Q replacing K were consistent with those of the lactylated proteins generated by the site‐specific lactylation system. As shown in our EMSA data, the SP1‐K694Q mutant exhibited significantly enhanced DNA‐binding affinity compared to SP1‐WT, which provided functional evidence in our study to strengthen the validity of the K694Q mutant. Second, while we have identified ACAT1 as a putative lactyltransferase regulator for SP1‐K694, we did not perform in vivo intervention experiments using ACAT1‐specific inhibitors (e.g., Avasimibe) in COPD models, and thus the therapeutic efficacy of targeting this pathway remains to be validated. ACAT1 possesses dual enzymatic activities as both an acetyltransferase and a lactyltransferase. How ACAT1 switches its substrate preference between acetylation and lactylation under different metabolic states remains an open question and needs future investigation. Third, our human lung tissue cohort did not distinguish between acute exacerbation and stable COPD patients, which may have precluded a thorough delineation of the relationship between the relevant indicators and disease activity. This issue will be addressed in subsequent investigations. We acknowledge that the sample size of our human lung tissue cohort is relatively modest. Nevertheless, the significant association between TREM1 expression and lung function parameters (FEV1, FEV1%pred, and FEV1/FVC) remained robust after adjusting for age, sex, BMI, and pack‐years. This finding, consistent with our in vitro and in vivo mechanistic data, supports the biological plausibility of this association and needs validation in larger independent cohorts. From a translational perspective, the development of cell Triggering receptor expressed on myeloid cells 1membrane‐associated liposomes and biomimetic nanocarriers offers promising strategies for targeted therapeutic delivery [46]. They could be engineered to deliver ACAT1 inhibitors or TREM1‐targeting agents specifically to alveolar macrophages, offering a targeted therapeutic strategy for COPD.

## 4 Conclusion

Collectively, this study identifies a novel mechanism linking lactylation to macrophage dysfunction in COPD. We show that elevated LDHA and ACAT1 expression in macrophages promotes SP1‐K694 lactylation, which in turn facilitates TREM1 transcription and drives M1 polarization, culminating in airway inflammation. Mechanistically, these findings define the LDHA/ACAT1‐SP1‐TREM1 axis as a potential therapeutic target and elucidate the role of non‐histone lactylation in COPD.  From a translational perspective, leveraging existing advanced delivery technologies such as cell membrane‐biomimetic nanoparticles could enable targeted delivery of ACAT1 or TREM1 modulators to alveolar macrophages, offering a promising therapeutic strategy for COPD

## 5 Methods

### 5.1 Isolation and Culture of Bone Marrow‐Derived Macrophages (BMDMs)

BMDMs were isolated from 6‐ to 8‐week‐old C57BL/6J mice purchased from SJA Lab Animals Corporation (Hunan, China). Femurs and tibiae were collected under sterile conditions, and bone marrow was flushed with Dulbecco's Modified Eagle Medium (8122386; Gibco, USA) containing 10% heat‐inactivated fetal bovine serum (10100147; Gibco, USA) and Primocin (ant‐pm‐05; InvivoGen, France). After passing through a 70µm cell strainer (352350; Falcon, Japan) and red blood cell lysis (P00138; Beyotime, China), cells were seeded in culture dishes (Saining, China) and cultured in the same medium supplemented with 20 ng/mL recombinant murine macrophage colony‐stimulating factor (Novoprotein, China). Fresh medium containing macrophage colony‐stimulating factor was added on day 3, and the medium was completely replaced on day 6. On day 7, adherent BMDMs were harvested using TrypLE (Gibco, USA). Purity (>90%) was confirmed by flow cytometry using antibodies against F4/80 and CD11b.

### 5.2 Animal Models and Treatments

In Experiment 1, male C57BL/6J mice, Lyz2^em1Cin(iCre)+/−^ (abbreviated as Lyz2^iCre+/−^) mice, and TREM1^loxp/wt^ mice used in this study were designed and generated by GemPharmatech Co., Ltd. (China). By breeding Lyz2^iCre+/−^ and TREM1^loxp/wt^ mice, littermate male mice with genotypes Lyz2^iCre+/−^TREM1^loxp/loxp^ (conditional knockout [CKO] mice) and Lyz2^iCre−/−^TREM1^loxp/loxp^ (wild‐type [WT] mice) were obtained for subsequent experiments. Genomic DNA was extracted from mouse tail biopsies using DirectPCR (Tail) solution (102‐T; Viagen Biotech Inc., USA). Genotyping was performed by Polymerase Chain Reaction(PCR amplification of the Lyz2 and Trem1 loci. PCR products were separated by 2% agarose gel electrophoresis at 140 V for 30 min and visualized using a ChemiDoc MP Imaging System (Bio‐Rad). BMDMs were isolated from WT and CKO mice, and TREM1 mRNA and protein expression were assessed by qPCR and western blotting to confirm myeloid‐specific TREM1 deletion.

To establish the COPD mouse model, mice were subjected to cigarette smoke exposure using an automated animal smoking apparatus (Tow‐Int‐Tech, China). Exposure was performed for 12 consecutive weeks, 5 days per week, with 2 sessions per day. In each session, mice were exposed to smoke generated from eight cigarettes over a 1‐h period.

In Experiment 2, we investigated the impact of lactylation on macrophage polarization and COPD. Mice were assigned to three groups: control, COPD, and oxamate+COPD. Mice in the oxamate+COPD group received an intraperitoneal injection of sodium oxamate (500 mg/kg, S6871; Selleck, China) or sodium L‐lactate (500 mg/kg, L7022; Sigma–Aldrich) for 14 consecutive days before cigarette smoke exposure.

In Experiment 3, to explore the role of SP1‐K694la in COPD, the lysine (K) at position 694 of SP1 was replaced with arginine (R) to mimic delactylation, or with glutamine (Q) to mimic constitutive lactylation. The corresponding adeno‐associated virus (AAV) constructs, designated AAV‐SP1‐K694R and AAV‐SP1‐K694Q, were generated and packaged by Genechem (Shanghai, China). Mice were assigned to the following groups: control, COPD, AAV‐NC‐COPD, AAV‐SP1‐K694R‐COPD, and AAV‐SP1‐K694Q‐COPD. After 2 weeks of cigarette smoke exposure, AAV‐SP1‐K694R, AAV‐SP1‐K694Q, and AAV‐NC were administered via intratracheal injection at a dose of 1.5 × 10^11^ vg per mouse in the respective groups. The viral vectors were diluted in sterile phosphate‐buffered saline to a final volume of 50 µL and delivered using a microsprayer.

### 5.3 Lung Function Measurements

Pulmonary function was assessed using a small animal testing system (PLY3211, Buxco Electronics, USA) following previously described protocols [47]. Pulmonary function was assessed 24 h after the final cigarette smoke exposure. Briefly, mice were anesthetized with an intraperitoneal injection of pentobarbital sodium (50 mg/kg), followed by tracheostomy. The tracheal cannula was then connected to a computer‑controlled spirometer. Measurements included forced expiratory volume in 100 ms (FEV100), peak expiratory flow (PEF), Lung resistance (RL) and the ratio of inspiratory time to expiratory time (TI/TE). Following the procedure, mice were euthanized by cervical dislocation.

### 5.4 Histological Analysis, Immunohistochemistry (IHC), and Immunofluorescence (IF)

The left lung was fixed in 4% paraformaldehyde, embedded in paraffin, sectioned at 4 µm thickness, and stained with hematoxylin and eosin (H3136, Sigma, USA). Mean linear intercept (MLI) and destructive index (DI) were measured as previously described [6]. Airway inflammation was also evaluated. IHC was conducted by incubating sections with primary and secondary antibodies at 4°C. The immunoreaction was visualized with an Horseradish Peroxidase(HRP) conjugated polymer. For IF analysis, MH‐S cells or frozen lung sections of mice and patients were fixed in 4% paraformaldehyde (20 min), permeabilized with 3% Triton X‑100 (30 min, room temperature), and blocked with 10% goat serum (30 min). Samples were incubated overnight with primary (F4/80 (81668‐1‐RR, Proteintech, USA), CD68 (28058‐1‐AP, Proteintech, USA), TREM1 (11791‐1‐AP, Proteintech, USA) and fluorescent secondary antibodies, followed by DAPI nuclear staining. Positive cells were visualized and quantified using a Zeiss microscope. Antibodies used: F4/80 (#30325, Cell signaling technology, USA), CD68 (#26042, Cell signaling technology, USA), CD206(#87887, Cell signaling technology, USA), TREM1 (AF1187, R&D Systems, USA) and Pan Kla (PTM‐1401RM, PTM BIO, China).

### 5.5 Flow Cytometry

Single‑cell suspensions from mouse lung tissue or MH‐S cells were centrifuged at 400  ×  g for 10 min at 4°C. Cells were then stained with a fixable viability dye(65‐0866, Invitrogen, USA) to discriminate live cells, followed by Fc receptor blocking(E‐AB‐F0997A, Elabscience, China) to reduce non‑specific binding. For lung tissue‑derived cells, surface staining was performed with these five antibodies against macrophage markers: CD45 APC‐Cy7(56103, BD Pharmingen, USA), CD11b FITC (561688, BD Pharmingen, USA), F4/80 PE(565410, BD Pharmingen, USA), CD86 PE‐Cy7(560582, BD Pharmingen, USA), and CD206(568809, BD Pharmingen, USA). For MH‐S cells, surface staining with anti‐CD86(123107, Biolegend, USA) antibody was performed first. Subsequently, cells were fixed and permeabilized using a fixation/permeabilization kit, followed by intracellular staining with anti‐CD206(141707, Biolegend, USA) antibody.

### 5.6 Cell Culture and Establishment of Stable Cell Lines Overexpressing SP1‐WT, SP1‐K694R, and SP1‐K694Q

MH‐S cells (MD159) were purchased from Sencells and cultured in RPMI‐1640 medium (Gibco, USA) supplemented with 10% fetal bovine serum, penicillin (100 µg/mL), and streptomycin (100 µg/mL) in a humidified atmosphere of 5% carbon dioxide at 37°C. To explore the role of SP1‐K694la in CSE‐induced MH‐S cells, the lysine (K) at position 694 of SP1 was replaced with arginine (R) to mimic delactylation, or with glutamine (Q) to mimic constitutive lactylation. SP1‐K694R and SP1‐K694Q constructs were generated and packaged into lentiviral vectors (Lenti‐SP1‐WT, LV‐SP1‐K694R and LV‐SP1‐K694Q) by Genechem (Shanghai, China). For stable transduction, MH‐S cells were seeded in six‐well plates at a density of 5 × 10^5^ cells per well and infected with concentrated lentivirus at a multiplicity of infection of 50. After 16 h, the medium was replaced with fresh culture medium. Stable transductants were selected with puromycin (4 µg/mL) for 2 days. Overexpression of SP1 and the presence of K694R or K694Q mutations were confirmed by western blotting using anti‐SP1 and anti‐FLAG antibodies. The K694Q mutant was generated to mimic lysine lactylation, as glutamine substitution has been extensively employed to study the functional consequences of lysine acylation modifications. Although K→Q mutagenesis does not perfectly replicate the chemical properties of lactyl‐lysine, it remains the most widely accessible genetic approach for initial functional characterization of lysine modifications. All mutant phenotypes were interpreted with this limitation in mind and validated by orthogonal approaches where possible.

### 5.7 siRNA Transfection and Cell Treatment

The siRNA targeting TREM1 (si‐TREM1) and the negative control siRNA (si‐NC) were designed and synthesized by RiboBio Corporation (Guangzhou, China). MH‐S cells were cultured in six‐well plates (NEST Biotechnology, China) to 70%–80% confluence prior to transfection. Transfection was carried out using Lipofectamine 3000 (L3000015; Invitrogen, USA) according to the manufacturer's instructions. At 48 h post‐transfection, cells were exposed to CSE for subsequent experiments.

### 5.8 Sample Preparation and PTM Enrichment

Cells were lysed in urea lysis buffer (8 m urea, 50 mm Tris‐HCl pH 8.0, 75 mm NaCl) supplemented with protease inhibitor cocktail (Merck Millipore) and deacetylase inhibitors (MedChemExpress). Protein concentration was determined using a Bicinchoninic Acid (BCA) assay (Thermo Fisher Scientific). For total proteome analysis, proteins were reduced, alkylated, and digested with trypsin (1:50, enzyme‐to‐protein ratio) at 37°C overnight. For lactylated peptide enrichment, tryptic peptides were dissolved in NETN buffer (100 mm NaCl, 1 mm EDTA, 50 mm Tris‐HCl, 0.5% NP‐40, pH 8.0) and incubated with pre‐washed anti‐lactyllysine antibody beads (PTM Bio) at 4°C overnight with gentle shaking. Bound peptides were eluted and desalted using C18 ZipTips (Millipore) prior to LC‐MS/MS analysis.

### 5.9 LC‐MS/MS Analysis

LC‐MS/MS analysis was conducted at Jingjie PTM BioLabs (Hangzhou, China). Tryptic peptides were dissolved in solvent A (0.1% formic acid, 2% acetonitrile in water), separated on a homemade reversed‐phase column (25 cm × 100 µm i.d.) using a nanoElute UHPLC system (Bruker Daltonics), and analyzed on a timsTOF Pro mass spectrometer (Bruker Daltonics) in PASEF mode. MS/MS data were searched against the Mus_musculus_10090_SP_20231220.fasta (17191 entries) concatenated with a reverse decoy database. with FDR < 1%. Lysine lactylation sites with localization probability > 0.75 were retained. Relative quantitative values were obtained from two independent experiments, and lactylation peptide ratios were normalized to their corresponding protein expression levels. Pathway annotation and protein‐protein interaction analysis were performed using KEGG and STRING databases, respectively.

### 5.10 Western Blot Analysis and Immunoprecipitation

Mouse or human lung tissues and cells were lysed with lysis buffer (P0013B, Beyotime, China). For Western Blot analysis, the protein samples were quantified. The same amount of protein (20 µg) was separated by 10% SDS‐PAGE and transferred onto PVDF membranes (Millipore, MA, USA). After blocking with 5% skim milk, the membranes were probed with primary antibodies overnight at 4°C, followed by incubation with secondary antibodies for 1 h at room temperature. The signals were detected and then quantified with ImageJ software. T. WB for detecting GAPDH (10494‐1‐AP, Proteintech, USA, 1:5000), and β‐actin (AC038, ABclonal, China, 1:10000), LDHA(Proteintech), Pan‐Kla (PTM‐1401RM, PTM BIO), SP1(Abcam), TREM1(11791‐1‐AP, Proteintech, USA), SP1‐K694la (PTM Bio) and ACAT1(16215‐1‐AP, Proteintech) were performed. The Western blot was performed three times in technical and biological replicates.

For Co‐IP and lactylation immunoprecipitation, 30 µg protein lysate was used for input. 20 µlof prewashed beads were added to 1 mg of protein lysate and incubated at 4°C for 1 h with rotation. The beads were then separated from the lysate using magnetic separation, and the precleared lysate was transferred to a clean tube. Subsequently, 1 mg of protein lysate was incubated with primary antibodies against SP1, pan‐Kla, and ACAT1, respectively, at 4°C for 12 h, followed by co‐incubation with prewashed beads at 4°C for 2 h. After thorough washing, the bound proteins were detected by Western blot using the corresponding antibodies.

### 5.11 Lactate Measurement

The concentrations of human serum lactate, mouse serum lactate and cell lactate were measured using the Lactate Assay Kit (EBC‐K044M, Elabscience, China).

### 5.12 Chromatin Immunoprecipitation (ChIP) Assay

ChIP assay was performed using the SimpleChIP Plus Enzymatic Chromatin IP Kit (CST, 9004S). Briefly, MH‐S cells (1 × 10^7^) were crosslinked and resuspended in ChIP buffer supplemented with 1 × Protease Inhibitor Cocktail (CST). Chromatin was digested with micrococcal nuclease to generate DNA fragments of approximately 150–900 bp. Immunoprecipitation was carried out by incubating the digested chromatin overnight at 4°C with anti‑SP1 (CST, 46395S) or control anti‑IgG (CST, 2729) antibodies, followed by capture with protein A/G agarose beads. After washing and elution, crosslinking was reversed, and DNA was purified. Primers targeting the positive region were designed to flank the predicted three SP1‐binding motifs within the TREM1 promoter. To verify specificity, a negative control primer pair was designed to amplify a distal region within the TREM1 promoter that lacks any consensus SP1‐binding sequences (based on bioinformatic prediction). SP1 occupancy at the TREM1 promoter was assessed by quantitative real‑time PCR (ChIP‑qPCR). The primer sequences are listed in Table S6.

### 5.13 Electrophoretic Mobility Shift Assay (EMSA)

EMSA was conducted with the LightShift Chemiluminescent EMSA Kit (Thermo Fisher Scientific) according to the recommended protocol. Purified recombinant SP1 proteins (WT, K694Q, or K694R) at 100 ng per reaction were mixed with 20 fmol of Cy5.5‐labelled double‐stranded DNA probe containing the SP1 consensus binding sequence, in binding buffer supplemented with 50 ng/µl poly(dI‐dC) as a nonspecific competitor. After incubation for 20 min at room temperature, the reaction mixtures were separated on a native 6% polyacrylamide gel (100 V, 60 min) and electrotransferred to nylon membranes. Chemiluminescent detection was performed using the supplied detection module. For competition experiments, a 100‐fold molar excess of unlabeled wild‐type or mutant probe was co‐incubated with the recombinant protein prior to electrophoresis.

### 5.14 In Vitro Lactylation Assay

Recombinant ACAT1 (100 ng, URPD664Mu01, Cloud‐Clone group, Wuhan) was incubated with Flag‐SP1(200 ng) in reaction buffer (50 mm HEPES pH7.5, 150 mm NaCl, 0.25 mm EDTA, 5 mm MgCl, 1 mm DTT) containing 100 µm lactyl‐CoA (TargetMol) at 37°C for 2 h. Reactions were quenched with 5 × SDS buffer, denatured at 100°C for 10 min. Samples were resolved by SDS‐PAGE and subjected to immunoblotting using specified antibodies to detect lactylation modifications.

### 5.15 ELISA

We collected the plasma samples from COPD patients and control participants. And collected BALF from COPD and control mice. The concentration of TREM1 in human plasma was detected by ELISA kits (JL11751, Jonlnbio, China), and the concentration of IL‐1β, IL‐6, TNF‐α in BALF and cell supernatant was detected by ELISA kits (JL18442, JL20268, JL10484, Jonlnbio, China) according to the instructions.

### 5.16 Reverse Transcription Quantitative Polymerase Chain Reaction (RT‐qPCR)

Total RNA was isolated using TRIzol reagent (BS258A, Biosharp, China). First‑strand cDNA was synthesized with the RevertAid First Strand cDNA Synthesis Kit (K1622, Thermo Fisher Scientific, USA). Quantitative real‐time PCR (RT‐qPCR) was subsequently carried out using SYBR Green master mix (11184ES08, Yeasen, China) according to the manufacturer's protocol. The primers for Human TREM1: Forward, catgcacagagaggccttca; Reverse, agtggagacatcggcagttg. The primers for Mouse TREM1: Forward, tgctgtgcgtgttctttgtc; Reverse, cactgtcagcatctgtccca.

### 5.17 Statistical Analyses

Data are presented as mean ± standard error of the mean (SEM). Replicate numbers for each experiment and results of the statistical analyses are mentioned in the Figure legends. Two‐sided unpaired Student's *t* test was employed to compare two groups, while one‐way analysis of variance (ANOVA) followed by Tukey's post hoc test was used for multiple groups. P values are indicated in the Figures. *p*<0.05 was considered statistically significant. All statistical analyses were performed with SPSS version 27.0 and Free Statistics version 1.7.1, and graphs were generated with GraphPad Prism 9.5.0.

## Author Contributions

**L.L**. was responsible for the majority of experimental work, statistical analysis, and the original manuscript preparation. Partial experiments were completed by **P.Z., Q.S., T.L., P.Z., and Y.Q. Z. P.C**. designed and oversaw the project and contributed to manuscript editing. All authors approved the final version, consented to the journal of submission, and accept accountability for every part of the study.

## Funding

This work was supported by grants from the National Natural Science Foundation of China (NSFC, 82270045 and 82470037 to **Ping Chen**).

## Ethics Statement

Human study: Title of the approved project: YY1 lactylation regulates TREM1 in alveolar macrophages and mediates apoptosis of pulmonary endothelial cells in COPD; This study was approved by the Ethics Committee of the Second Xiangya Hospital of Central South University (Number: Z0208‐01); Date: March 7, 2024. All patients were provided written informed consent.

Animal study: Title of the approved project: YY1 lactylation regulates TREM1 in alveolar macrophages and mediates apoptosis of pulmonary endothelial cells in COPD; This study was approved by the Ethics Committee of the Second Xiangya Hospital of Central South University (Number: 20240124); Date: January 24, 2024.

## Consent

The authors have nothing to report.

## Conflicts of Interest

The authors declare no conflicts of interest.

## Supporting information




**Supporting File 1**: advs78021‐sup‐0001‐SuppMat.docx.

**Supporting File 2**: advs78021‐sup‐0002‐SuppMat.docx.

## Data Availability

The sequencing data reported in this study are not yet publicly available now. They will be deposited in ProteomeXchange prior to publication. Accession numbers will be provided upon data release.
