# Supplementary material for: ACAT1‐Mediated SP1‐K694 Lactylation Promotes M1 Macrophage Polarization via TREM1 Transcription in COPD
Source: Adv Sci (Weinh). 2026 Sep 27:e78021. Online ahead of print. doi: 10.1002/advs.78021 (PMC13616354; doi:10.1002/advs.78021)
Supplement: Supplementary file 1 [file ADVS-9999-e78021-s001.docx]

**Supplemental Table 1. The clinical characteristics of the patients.**

| **Variables** | **Control participants**  **(n=10)** | **COPD patients**  **(n=23)** | ***p -***  **value** |
| --- | --- | --- | --- |
| Age (years) (Mean ± SD) | 59.3±9.7 | 60.2±7.3 | 0.779 |
| Sex, n (%) |  |  | 0.582 |
| Male | 6 (60.0) | 13 (56.5) |  |
| Female | 4 (40.0) | 10 (43.5) |  |
| BMI (kg/m^2^) (Mean ± SD) | 23.6±4.2 | 22.8±4.0 | 0.631 |
| Smoking (pack/year) (Median, IQR) | 0 (30) | 22.5(42.0) | **＜0.001** |
| Pulmonary function (Mean ± SD) |  |  |  |
| FEV1 | 2.3±0.6 | 1.5±0.5 | **0.021** |
| FEV1 %pred | 81.4±18.3 | 57.3±13.6 | **0.008** |
| FEV1/FVC | 81.7±19.5 | 55.6±11.2 | **0.005** |
| Lactate in whole blood(mmol/L) | 1.12±0.39 | 1.86±0.629 | **0.001** |

**Abbreviations:** BMI, Body mass index; COPD, Chronic obstructive pulmonary disease; FEV1, Forced expiratory volume in one second; FVC, Forced vital capacity.The bold p value indicate statistical significance.

**Supplemental Table 2. The demographic data and clinical characteristics of the patients.**

| **Variables** | **Control participants**  **(n=15)** | **COPD patients**  **(n=13)** | ***p -***  **value** |
| --- | --- | --- | --- |
| Age (years) (Mean ± SD) | 58.4±1.1 | 58.8±10.6 | 0.929 |
| Sex, n (%) |  |  | 0.396 |
| Male | 10 (66.7) | 9 (69.2) |  |
| Female | 5 (33.3) | 4 (30.8) |  |
| BMI (kg/m^2^) (Mean ± SD) | 24.0±2.7 | 23.6±2.0 | 0.677 |
| Smoking (pack/year) (Median, IQR) | 10 (20.5) | 30(25.0) | **0.015** |
| Pulmonary function (Mean ± SD) |  |  |  |
| FEV1 | 2.4±0.6 | 1.6±0.5 | **0.028** |
| FEV1 %pred | 96.9±21.2 | 61.0±13.6 | **0.002** |
| FEV1/FVC | 79.6±15.1 | 56.7±12.6 | **0.002** |

**Abbreviations:** BMI, Body mass index; COPD, Chronic obstructive pulmonary disease; FEV1, Forced expiratory volume in one second; FVC, Forced vital capacity.The bold p value indicate statistical significance.

**Supplemental Table 3. Multivariable linear regression analysis of the association between lactate level and clinical features in human serum.**

|  | **OR (95% CI)** | *P*-  value |
| --- | --- | --- |
| Smoking index | 1.132(1.002-2.318) | 0.022 |
| GOLD grade |  |  |
| GOLD 1 | Reference |  |
| GOLD 2 | 1.083(0.924-2.347) | 0.314 |
| GOLD 3 | 1.179(1.002-1.905) | 0.032 |
| GOLD 4 | 1.215(1.004-1.942) | 0.026 |

**Notes:**Variables in logistic regression model:age, sex, BMI, GOLD grade and Smoking index. The bold p value indicate statistical significance.The bold p value indicate statistical significance.

**Supplemental Table 4. Multivariable linear regression analysis of the association between TREM1 level and clinical features.**

|  | **OR (95% CI)** | *P*-  value |
| --- | --- | --- |
| Smoking index | 1.218(1.007-1.833) | 0.008 |
| GOLD grade |  |  |
| GOLD 1 | Reference |  |
| GOLD 2 | 1.116(0.893-2.026) | 0.117 |
| GOLD 3 | 1.226(1.003-2.341) | 0.027 |
| GOLD 4 | 1.305(1.011-2.487) | 0.015 |

**Notes:**Variables in logistic regression model:age, sex, BMI, GOLD grade and Smoking index. The bold p value indicate statistical significance. The bold p value indicate statistical significance.

**Supplemental Table 5. Multivariable linear regression analysis of the association between TREM1 expression and pulmonary function in human lung.**

|  | β (95% CI) | *P*-  value |
| --- | --- | --- |
| FEV1 | -0.131  (-0.312—0.068) | 0.039 |
| FEV1 %pred | -0.246  (-0.392—-0.137) | 0.011 |
| FEV1/FVC | -0.241  (-0.388—-0.145) | 0.009 |

**Notes:**Variables in linear regression model: age, sex, BMI, FEV1, FEV1 %pred, FEV1/FVC and Smoking index. The bold p value indicate statistical significance.

| **Supplemental Table 6. Primers used in this study.** | |
| --- | --- |
| **qRT-PCR Primers** | |
| **Human** |  |
| TREM1 | 5′- CATGCACAGAGAGGCCTTCA -3′(forward) |
|  | 5′- AGTGGAGACATCGGCAGTTG -3′(reverse) |
| GAPDH | 5′-GTCTCCTCTGACTTCAACAGCG′(forward) |
|  | 5′-ACCACCCTGTTGCTGTAGCCAA′(reverse) |
| **Mouse** |  |
| TREM1 | 5′- TGCTGTGCGTGTTCTTTG-3′(forward) |
|  | 5′- CACTGTCAGCATCTGTCCC-3′(reverse) |
| ACAT1 | 5′-GCAGGGAAGTTTGCCAGTGAGA-3′(forward) |
|  | 5′-GAACACGGTCTTGAGCTTTGGC-3′(reverse) |
| β-actin | 5′-GAGACCTTCAACACCCCAGC-3′(forward) |
|  | 5′-ATGTCACGCACGATTTCCC-3′(reverse) |
| **ChIP-qPCR Primers** | |
| TREM1-prom-F1 | GGCCTCTGTCTCAATTACTCAGGAG |
| TREM1-prom-R1 | TGGGACCTCAGTTTCCTCTTCTCTG |
| TREM1-prom-F2 | AGGGACCACAGGGAGAAAGAAGAG |
| TREM1-prom-R2 | GGCTGCTTCTGCTGTAACTGTCTG |
| TREM1-prom-F3 | TGTGTATGTGGGCAAATGTAGTG |
| TREM1-prom-R3 | TTTGGCCATGATGTTTGTGC |
| **CKO-mice identification Primers** | |
| Lyz-F | 5′-AGTGCTGAAGTCCATAGATCGG-3′(forward) |
| Lyz-R | 5′-CTGATTCTCCTCATCACCAGG-3′(forward) |
| Flox(TREM1)-F | 5′-GAATAGCCAGAGAACAGGTGGC-3′(forward) |
| Flox(TREM1)-R | 5′-CTGCCTGTCTGGCATCATTTC-3′(forward) |
