# Supplementary material for: ACAT1‐Mediated SP1‐K694 Lactylation Promotes M1 Macrophage Polarization via TREM1 Transcription in COPD
Source: Adv Sci (Weinh). 2026 Sep 27:e78021. Online ahead of print. doi: 10.1002/advs.78021 (PMC13616354; doi:10.1002/advs.78021)
Supplement: Supplementary file 2 [file ADVS-9999-e78021-s002.docx]

**Supplementary Figure 1. Lactylation levels are associated with macrophage polarization in COPD model mice.**

(A) Flow cytometry analysis of CD11b⁺F4-80⁺macrophages in BMDM cells. (B)The body weight of COPD model mice treated with L-Lactate and Sodium Oxamate(N=8 mice per group). (C) The lung function including FEV100, PEF , RL and TI/TE of the COPD model mice treated with L-Lactate and Sodium Oxamate(N=8 mice per group). (D) IF staining of Pan-Kla in macrophage in mice lung tissue of the COPD model mice treated with L-Lactate and Sodium Oxamate. Representative images of lung sections co-stained with anti-F4/80 (green, macrophage marker), anti-Pan-Kla (red) and Nuclei were counterstained with DAPI (blue)(scale bar = 100 µm)(N=8 mice per group). (E) IF staining of INOS and CD206 in macrophage in mice lung tissue of the COPD model mice treated with L-Lactate and Sodium Oxamate, Representative images of lung sections co-stained with anti-F4/80 (green, macrophage marker), anti-INOS (red), anti-CD206(pink) and Nuclei were counterstained with DAPI (blue)(scale bar = 100 µm).N=8 mice per group *p<0.05; **p<0.01; ***p<0.001.

**Supplementary Figure 2. SP1 K694la was increases in the CSE-induced macrophage.**

(A) Number of total identified peptides and lactylated peptides. (B) Quality control revealing that the distribution of peptides was in a reasonable range. (C) Functional classification of identified lactylated proteins. (D) Lactylated lysine residues were highlighted (red) in alignment of sequences surrounding K694 in SP1 homologs from diverse species.(E) Colocalization of SP1 and Pan-Kla in MH-S cells, which were co-stained with anti-Pan-Kla (green) and anti-SP1 (red). Nuclei were counterstained with DAPI (blue)(scale bar = 100 µm). (F) The expression of SP1 in CSE-induced MHS cells. (G) The expression of Pan-kla in CSE-induced MH-S cells.(H)The level of SP1 lactylation in CSE-induced MH-S cells was detected by IP using being immunoprecipitated with SP1 antibody or control IgG.(I)CD86 and CD206 expression in CSE-induced MH-S cells, detected by flow cytometry.. *p<0.05; **p<0.01; ***p<0.001.

**Supplementary Figure 3: SP1-K694Q mutation increased M1 macrophage polarisation in CSE-induced MH-S cells.**

(A) SP1 lactylation levels detected by IP in CSE-induced MH-S cells expressing the SP1-K694Q mutant. (B) CD86 and CD206 expression detected by flow cytometry, and (C) iNOS (green) and CD206 (orange) expression detected by IF (scale bar = 100 µm) in CSE-induced MH-S cells expressing the SP1-K694Q mutant. (D) Concentrations of IL-1β, IL-6, and TNF-α in the supernatant of CSE-induced MH-S cells after transduction with the SP1-K694Q mutant. (E) SP1-K694 lactylation levels detected by western bloting using a site-specific lactylation antibody (SP1-K694la) in CSE-induced MH-S cells expressing the WT-SP1, SP1-K694 mutants. (F) Specificity of SP1-K694 site-specific lactylation antibodies was examined by dot blotting. (G)lactylation of SP1-K694 in WT-SP1, SP1-K694 mutants was determined by IP. *p < 0.05, **p < 0.01, ***p < 0.001.

**Supplementary Figure 4. SP1-K694 lactylation induced M1 macrophages polarization by regulating TREM1 expression in COPD mice**

(A) The lung function including FEV100, PEF , RL and TI/TE of the COPD model mice intratracheal injected with AAV-SP1-K694R, AAV-SP1-K694Q and AAV-NC(n=8 mice per group).(B) Potential SP1 binding sites of TREM1 promoter. (C) ChIP-qPCR analysis was performed with primers spanning predicted TREM1 promoter sequences. (D)The expression of TREM1 in CSE-induced MH-S cells. (E)Recombinant SP1-WT, SP1-K694Q, and SP1-K694R proteins were incubated with biotin-labeled probe containing the SP1-binding motif from the TREM1 promoter. Cold competition was performed with 100-fold molar excess of unlabeled WT probe or mutant probe (SP1-binding motif disrupted). (F)The expression of TREM1 in Control mice and COPD model mice(n=8 mice per group). (G)The expression of SP1 K694la in Control mice and COPD model mice (n=8 mice per group).

**Supplementary Figure 5. Airway inflammation in Control mice and AAV-NC mice.** (A)TREM1expression in lung macrophages of COPD mice administrated with SP1 K694Q or K694R mutation. TREM1expression in lung macrophages of detected by IF. Representative images show lung sections co-stained with anti-F4/80 (green, macrophage marker), anti-TREM1 (red) in different groups (scale bar = 100 µm) (n=8 mice per group). (B) H&E staining of mouse lung tissue (scale bar = 200 µm). (C)The expression of TREM1 and SP1-K694la in Control mice and AAV-NC mice. (D) CD86 and CD206 expression in lung macrophages detected by flow cytometry, (E) iNOS and CD206 expression detected by IF. Representative images show lung sections co-stained with anti-F4/80 (green, macrophage marker), anti-iNOS (red), or anti-CD206 (pink) in different groups (scale bar = 100 µm). n = 8 mice per group;(F) Concentrations of IL-1β, IL-6, and TNF-α in BALF. n = 8 mice per group; *p<0.05; **p<0.01; ***p<0.001.

**Supplementary Figure 6. TREM1 overexpression rescued the SP1-K694R mutation-induced reduction in the M1 macrophage polarization in CSE-treated MH-S cells.** (A) Protein expression of SP1-SP1-K694la and TREM1 in CSE-induced MH-S cells transfected with OE-TREM1 and overexpressing the SP1-K694R mutant via lentiviral transduction. (B) CD86 and CD206 expression detected by flow cytometry, (C) iNOS (green) and CD206 (orange) expression detected by IF (scale bar = 100 µm), and (D) concentrations of IL-1β, IL-6, and TNF-α in the cell supernatant in CSE-induced MH-S cells transfected with OE-TREM1 and overexpressing the SP1-K694R mutant.

**Supplementary Figure 7: Validation of TREM1 knockout efficiency in macrophages and the M1 macrophages in CKO-COPD mice.**

Validation of si-TREM1 knockdown (A) at the mRNA level by RT-qPCR and (B) at the protein level by western blotting in MH-S cells. (C)Tail DNA electrophoresis for genotyping (D) The protein expression of TREM1 in BMDM cells isolated from WT mice and CKO mice. (E) The mRNA expression of TREM1 in BMDM cells isolated from WT mice and CKO mice. (F) The lung function including FEV100, PEF , RL and TI/TE of the COPD model mice and CKO-COPD mice (N=8 mice per group). (G) IF staining of INOS in macrophage in mice lung tissue. Representative images of lung sections co-stained with anti-F4/80 (green, macrophage marker), anti-INOS (red) and anti-CD206 (pink), Nuclei were counterstained with DAPI (blue)(scale bar = 100 µm)(N=8 mice per group). *p<0.05; **p<0.01; ***p<0.001.

**Supplementary Figure 8: IF staing the expression of TREM1 in lung macrophages in CKO-COPD mice administrated with SP1 K694Q mutation.**

IF staining of TREM1 in lung macrophage in mice lung tissue(D), Representative images of lung sections co-stained with anti-F4/80 (green, macrophage marker) and anti-TREM1 (red)(scale bar = 100 µm) in lung tissue of mice (scale bar = 100 µm)(N=8 mice per group).

**Supplementary Figure 9: Identification of ACAT1 as an SP1-K694 lactyltransferase.**

(A) The protein expression of DLAT and SP1 K694la in DLAT-overexpressed MH-S cells. (B)The protein expression of ACAT1 and SP1 K694la in ACAT1-overexpressed MH-S cells. (C) The protein expression of Pan-Kac in ACAT1-overexpressed MH-S cells. (D) Predicted three-dimensional interaction between SP1 and ACAT1 using HDOCK, with SP1 shown in blue and ACAT1 in green. (E) The protein expression of ACAT1 in CSE-treated MH-S cells. (E) The protein expression of ACAT1 in Control mice and COPD mice(N=8 mice per group). (G)Western blot analysis of ACAT1 expression MHS cells transfected with si-TREM1 and Lenti-SP1 K694Q. (H)The protein expression of ACAT1 in COPD model mice administrated with TREM1-CKO and SP1-K694Q mutation at the same time.

**Supplementary Figure 10:Knockdown of ACAT1 decreases SP1 k694 lactylation.**

(A)Western blot analysis of ACAT1 expression in MHS cells transfected with si-NC or si-ACAT1. (B) The protein expression of ACAT1, SP1 K694la and TREM1 in CSE-treated MH-S cells transfected with si-ACAT1. H-I. CD86 and CD206 expression detected by Flow cytometry (C) and INOS (Green)and CD206(Orange) expression detected by IF(scale bar = 100 µm) (D) in CSE-induced MHS cells transfected with si-ACAT1.*p<0.05; **p<0.01; ***p<0.001.

**Supplementary Figure 11: Relationships of serum lactate and TREM1 with smoking index and GOLD grades, and IF staining the expression of TREM1、CD86 and CD206 in lung macrophage in COPD patients.**

(A)Scatter plots showing the correlations of serum lactate levels with smoking index; (B) Comparison of serum lactate levels among COPD patients stratified by GOLD grades 1, 2, 3, and 4; (C) Scatter plots showing the correlations of serum TREM1 levels with smoking index; (D) Comparison of serum TREM1 levels among COPD patients stratified by GOLD grades 1, 2, 3, and 4; E-G.The expression of TREM1, CD86 and CD206 in the lung macrophages. IF staining of TREM1 in macrophage in human lung tissue(E), Representative images of lung sections co-stained with anti-CD68 (green, macrophage marker), anti-TREM1 (red) and Nuclei were counterstained with DAPI (blue)(scale bar = 100 µm); IF staining of CD86 in macrophage in human lung tissue(F), Representative images of lung sections co-stained with anti-CD68 (green, macrophage marker), anti-CD86 (red) and Nuclei were counterstained with DAPI (blue)(scale bar = 100 µm); IF staining of CD206 in macrophage in human lung tissue(G), Representative images of lung sections co-stained with anti-CD68 (green, macrophage marker), anti-CD206 (red) and Nuclei were counterstained with DAPI (blue)(scale bar = 100 µm) in control participants and COPD patient’s lung tissue; Control participants (N=15) and COPD patients (N=13).*p<0.05; ***p<0.001.
